# *PCSK9* deficiency promotes the development of peripheral neuropathy

**DOI:** 10.1172/jci.insight.183786

**Published:** 2025-05-08

**Authors:** Ali K. Jaafar, Aurélie Paulo-Ramos, Guillaume Rastoldo, Bryan Veeren, Cynthia Planesse, Matthieu Bringart, Philippe Rondeau, Kévin Chemello, Olivier Meilhac, Gilles C. Lambert, Steeve Bourane

**Affiliations:** 1 Université de La Réunion, INSERM, UMR 1188 Diabète athérothrombose Thérapies Réunion Océan Indien (DéTROI), Saint-Pierre, La Réunion, France,; 2 CHU de La Réunion, Saint-Pierre, France.

**Keywords:** Metabolism, Neuroscience, Cholesterol, Neurodegeneration, Pain

## Abstract

Proprotein convertase subtilisin/kexin type 9 (*PCSK9*) induces the hepatic degradation of the low-density lipoprotein receptor (*LDLR*), thereby increasing the concentration of LDL-cholesterol in the blood. Beyond its effects on LDL, recent studies have reported pleiotropic effects of *PCSK9*, notably in septic shock, vascular inflammation, viral infection, and cancer. While the functional and structural integrity of peripheral nerves are critically influenced by circulating lipids, the effect of *PCSK9* on the peripheral nervous system remains unknown. In this study, we investigated the consequences of *PCSK9* deficiency on peripheral nerves. We found that *PCSK9* deletion in mice leads to peripheral neuropathy, characterized by reduced thermal and mechanical pain sensations. *PCSK9*-deficient mice also presented with skin structural changes, including a reduction in nociceptive Schwann cell number, axonal swelling of Remak fibers, and hypomyelination of small nerve fibers. Interestingly, the peripheral nerves of *PCSK9*-deficient mice showed an upregulation of *CD36*, a fatty acid transporter, which correlated with increased nerve lipid content, structural mitochondrial abnormalities, and acylcarnitine accumulation. Our findings demonstrate that *PCSK9* plays a critical role in peripheral nerves by regulating lipid homeostasis and that its deficiency results in symptoms related to peripheral neuropathy.

## Abstract 2

The manuscript describe for the first time a physiological role of PCSK9 in the peripheral nervous system

## Graphical abstract

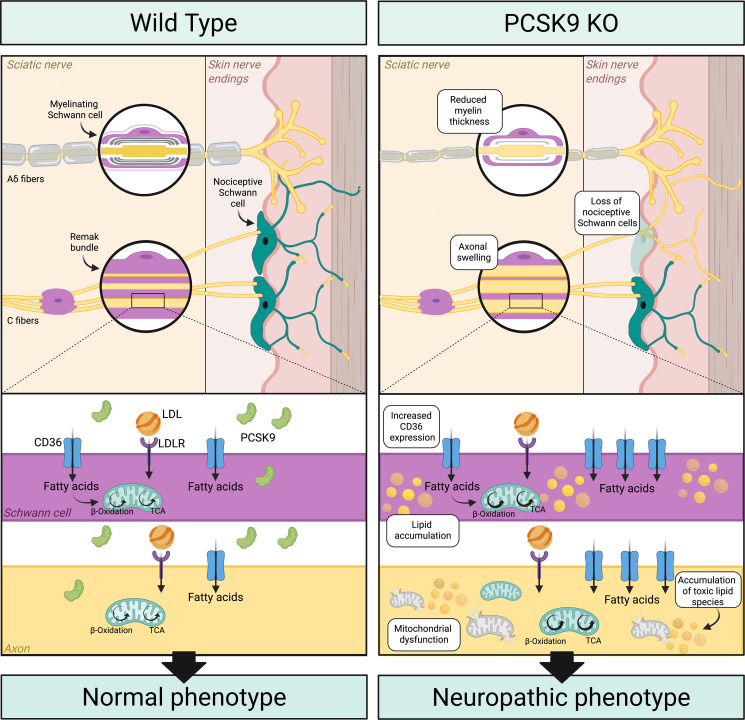

## Introduction

Proprotein convertase subtilisin/kexin type 9 (*PCSK9*) is a natural circulating inhibitor of the low-density lipoprotein receptor (*LDLR*) (1). It binds to the *LDLR* epidermal growth factor-A (EGFA) domain and targets it for lysosomal degradation, preventing *LDLR* recycling at the cell surface. As a result, *PCSK9* increases the circulating levels of LDL-cholesterol (LDL-C). *PCSK9* loss-of-function mutation carriers exhibit reduced LDL-C levels and are protected against atherosclerotic cardiovascular diseases (ASCVD) (2), whereas *PCSK9* gain-of-function mutation carriers present with familial hypercholesterolemia and are at high risk for ASCVD (3). These observations led to the successful development of *PCSK9* inhibitors, a new class of LDL-lowering drugs, prescribed on top of statins, which have demonstrated their efficacy in large cardiovascular outcome trials (4, 5). In addition, *PCSK9* also reduces the abundance of *LDLR*-related receptors, including the very low–density lipoprotein receptor (*VLDLR*), the *LDLR* related proteins 1 (*LRP1*) and 8 (*LRP8*, also known as *apoER2*), and the fatty acid transporter and scavenger receptor *CD36* in various tissues (1).

*PCSK9* is primarily expressed by the liver, and circulating *PCSK9* is almost exclusively of hepatic origin (6). However, the protein is also expressed in several other tissues — including the intestine, kidneys, and pancreas — and the nervous system (7, 8). *PCSK9* was initially cloned in cerebellar neurons undergoing apoptosis and originally called Neural Apoptosis-Regulated Convertase-1 (*NARC-1*) (7). In the central nervous system (CNS), *PCSK9* is expressed during cortical and cerebellar development, but its expression is restricted to the rostral extension of the olfactory peduncle in adult brain (7). *PCSK9* has also been detected in the cerebrospinal fluid of humans (9). There is evidence linking *PCSK9* to lipid homeostasis as well as neuronal differentiation and inflammation in the CNS (10). In the peripheral nervous system (PNS), *PCSK9* expression has been observed in a rat Schwann cell (SC) line (7) and in human tibial nerves at physiologically relevant levels (11). The roles of *PCSK9* in this tissue are still unknown. To date, there is no report of an association between *PCSK9* and peripheral nerve function. Only 3 individuals with total *PCSK9* loss of function have been reported in the literature, but none of them has been assessed for peripheral sensations. However, a small number of patients has reported thigh pain, weakness, and limb numbness following the initiation of *PCSK9* inhibitors treatment (12, 13).

Lipids play a key role in maintaining the structural and functional integrity of peripheral nerves (14, 15). In the PNS, free fatty acids, sphingolipids, glycerophospholipids, glycerolipids, and cholesterol are uniquely distributed across different cell types and cellular compartments (14, 16, 17). These lipids contribute to membrane structure, lipid rafts, organelles, and myelin sheath formation, as well as to signaling processes that regulate neuronal survival, growth, and synaptic transmission (18–21). Lipids are also a crucial energy source for nerve cells, which primarily use glucose but can also metabolize long-chain fatty acids via β-oxidation in mitochondria to produce ATP (22–24). Dysregulation of lipid metabolism can lead to altered sensory perception and neuropathy, with cholesterol playing an important role in inflammatory pain transmission (25) and in the functionality of the voltage-gated sodium channel *Nav1.7*, which is highly expressed in peripheral sensory neurons (26). Interestingly, low cholesterol levels appear to increase the risk of peripheral neuropathy in patients with type 2 diabetes (27, 28), and defects in lipid metabolism are present in other pathologies affecting the PNS, such as Fabry disease and Guillain-Barré syndrome (16, 29, 30).

Despite their capacity to endogenously synthesize lipids (31, 32), neurons (23) and SC (33) can take up lipids from the circulation, which is underlined by the abundant expression of lipoprotein receptors in the PNS. For instance, SC express the *LDLR*, *LRP1*, and *LRP8* (34–36). These receptors are also expressed in the dorsal root ganglia (DRG) along with the *VLDLR* (37). Given the importance of lipid homeostasis in peripheral nerves, the central role of *PCSK9* in lipid metabolism, and the abundant expression of its target receptors in this tissue, we hypothesized that *PCSK9* could play an important role in the PNS.

In this study, we demonstrate that *PCSK9* is expressed in peripheral sensory neurons and in SC. We found that *PCSK9* deficiency in mice significantly reduces their behavioral responses to thermal and mechanical pain stimuli. Additionally, *PCSK9*-deficient mice exhibit a marked increase in *CD36* expression and lipid content in peripheral nerves. These changes are accompanied by alterations in cellular lipid metabolism and energy production, including axonal swelling, mitochondrial dysfunction, and accumulation of acylcarnitines (AC), a toxic intermediate of fatty acid oxidation in the peripheral nerves.

## Results

### PCSK9 is expressed in the PNS.

**Figure 1:**
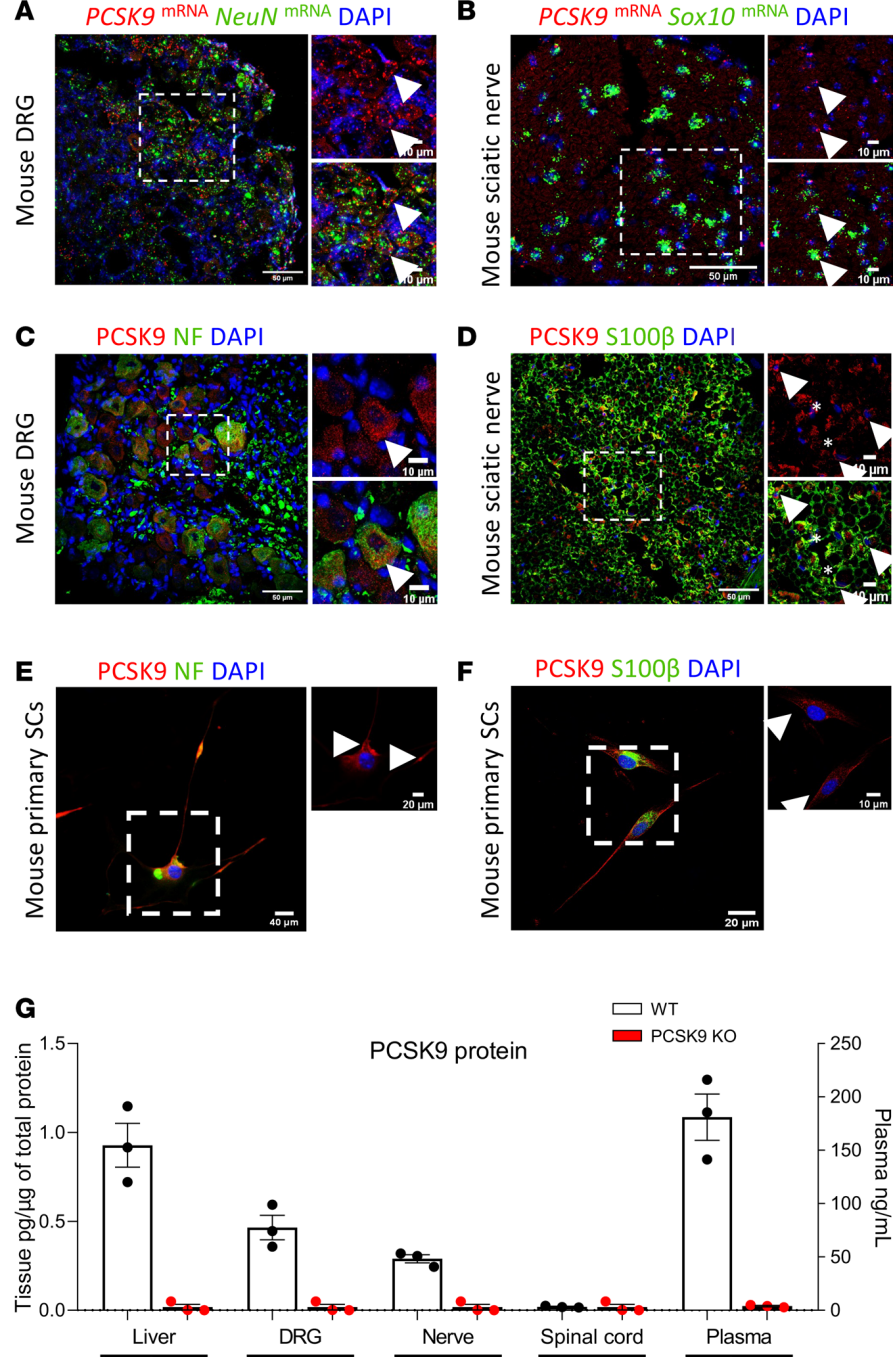
*PCSK9* expression in the peripheral nervous system of mice. (**A** and **B**) *PCSK9* mRNA expression in mouse dorsal root ganglia and sciatic nerve sections. Scale bars: main images (50 μm); insets (10 μm). (**C** and **D**) *PCSK9* protein expression in mouse dorsal root ganglia and sciatic nerve (arrowhead: *PCSK9* expression; asterisk: axonal *PCSK9* expression). Scale bars: main images (50 μm); insets (10 μm). (**E** and **F**) *PCSK9* expression in mouse primary sensory neurons and Schwann cells (arrowhead: *PCSK9* expression). Scale bars: main images (40 μm); insets (20 μm). (**G**) *PCSK9* protein concentration in liver, DRG, sciatic nerve, spinal cord and plasma in WT mice with the corresponding negative controls from *PCSK9-*KO mice; *n* = 3 mice per group. Data are shown as mean ± SEM. .

We assessed the expression of *PCSK9* in DRG and sciatic nerves of adult WT mice. Using multiplex in situ hybridization, we detected *PCSK9* mRNA transcripts in DRG neurons and sciatic nerve SC where they respectively colocalized with the neuronal marker *NeuN* and the SC marker *Sox10* (Figure 1, A and B). The specificity of *PCSK9* mRNA probes was confirmed in liver sections from WT and *PCSK9*-KO mice (Supplemental Figure 1, A and B; supplemental material available online with this article; https://doi.org/10.1172/jci.insight.183786DS1). Consistently, *PCSK9* protein was detected in both myelinated and unmyelinated mouse DRG neurons expressing Neurofilament (NF) (Figure 1C) and *CGRP/IB4*, respectively (Supplemental Figure 1C), in line with single-cell RNA-Seq datasets showing *PCSK9* expression in various sensory neuron subtypes (38). In sciatic nerves, *PCSK9* colocalized with both the pan-SC marker *S100β* (Figure 1D) and the nonmyelinating SC marker *L1CAM* (Supplemental Figure 1D). These observations were confirmed in primary sensory neurons and SC isolated from WT mice (Figure 1, E and F) and in human primary SC cultures (Supplemental Figure 1, E and F). Next, we quantified *PCSK9* protein levels in WT mice spinal cords, DRG, sciatic nerves, and liver extracts by ELISA, using *PCSK9*-KO mice as negative controls. We determined that, while *PCSK9* protein was undetectable in spinal cord protein extracts, significant levels were detected in the DRG (0.46 ± 0.11 pg/μg) and sciatic nerve (0.29 ± 0.03 pg/μg) extracts but at lower concentrations than those found in the liver (0.92 ± 0.11 pg/μg) (Figure 1G). These observations demonstrate that *PCSK9* is expressed in sensory neurons and SC within the PNS.

### PCSK9 deficiency impairs thermal and mechanical pain sensitivity.

**Figure 2:**
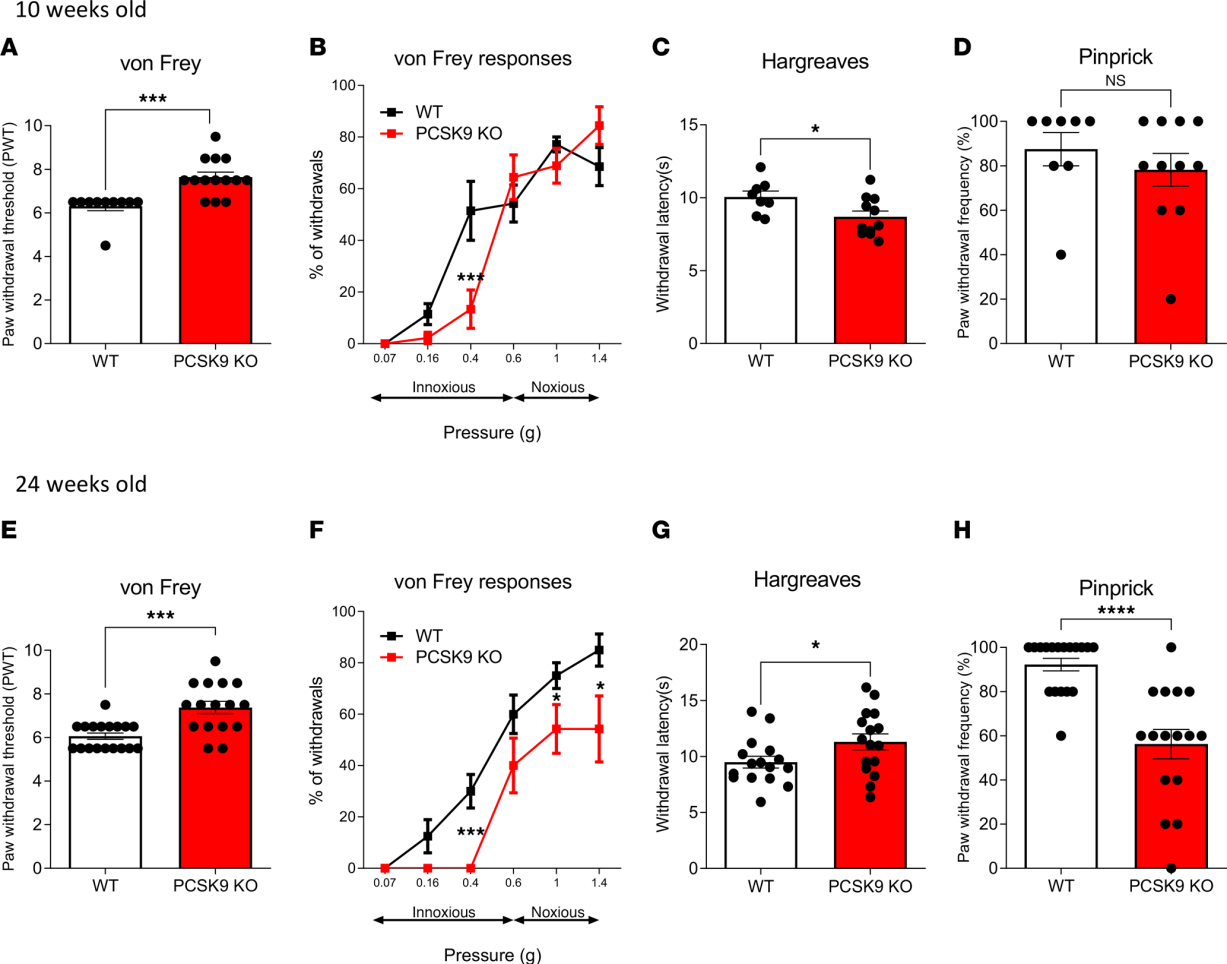
*PCSK9* deficiency is associated with sensory behavioral abnormalities. (**A** and **B**) Mechanical pain sensation evaluated with the von Frey test in 10-week-old WT (*n* = 10) and *PCSK9-*KO (*n* = 14) mice presented as paw withdrawal threshold and withdrawal percentage. (**C**) Thermal pain sensation evaluated with the Hargreaves test in 10-week-old WT (*n* = 8) and *PCSK9-*KO (*n* = 11) mice. (**D**) Acute mechanical pain sensation evaluated with the pinprick test in 10-week-old WT (*n* = 8) and *PCSK9-*KO (*n* = 11) mice. (**E** and **F**) Mechanical pain sensation evaluated with the von Frey test in 24-week-old WT (*n* = 18) and *PCSK9-*KO (*n* = 16) mice presented as paw withdrawal threshold and withdrawal percentage. (**G**) Thermal pain sensation evaluated with the Hargreaves test in 24-week-old WT (*n* = 16) and *PCSK9-*KO (*n* = 16) mice. (**H**) Acute mechanical pain sensation evaluated with the pinprick test in 24-week-old WT (*n* = 18) and *PCSK9-*KO (*n* = 16) mice. Data are expressed as means ± SEM and were analyzed by unpaired *t* test (**A**, **C**–**E**, **G**, and **H**) or 2-way ANOVA with Tukey’s multiple-comparison test (**B** and **F**). *P < 0.05, ***P < 0.001, and ****P < 0.0001.

To investigate the role of *PCSK9* in the PNS, we conducted a series of behavioral analyses to comparatively assess the sensory and motor acuity of WT and *PCSK9*-KO mice. At 10 weeks of age, *PCSK9*-KO mice exhibited reduced sensitivity to mechanical stimuli, evidenced by an increase in paw withdrawal threshold (PWT) and a decrease in the frequency of paw withdrawal in response to an innocuous stimulus with von Frey filaments (Figure 2, A and B). We also observed a significant increase in thermal pain sensitivity (i.e., a decrease in paw withdrawal latency) in *PCSK9-*KO mice compared with WT mice (Figure 2C). No significant differences were observed in their responses to noxious stimuli using either large-diameter von Frey filaments or the pinprick test (Figure 2, B and D). At 24 weeks of age, however, in addition to the reduction of light mechanical (Figure 2, E and F) and thermal pain (Figure 2G) sensations, *PCSK9-*KO mice also showed a significant reduction in responses to acute mechanical pain stimuli (Figure 2, F and H). Of note, *PCSK9-*KO mice at both ages did not show any significant impairment in response to dynamic touch sensation, nor did they exhibit impaired motor coordination or grip strength (Supplemental Figure 2, A, B, and D–F). In contrast, we failed to observe a significant sensory impairment in *PCSK9-*KO female mice (Supplemental Figure 2, C and G–I). Since males presented with a much stronger phenotype, we subsequently focused our analyses exclusively in male mice to understand how *PCSK9* deletion impairs thermal and mechanical pain sensations in vivo.

### PCSK9 deficiency impairs the nociceptive glio-neural complex within the epidermis.

**Figure 3:**
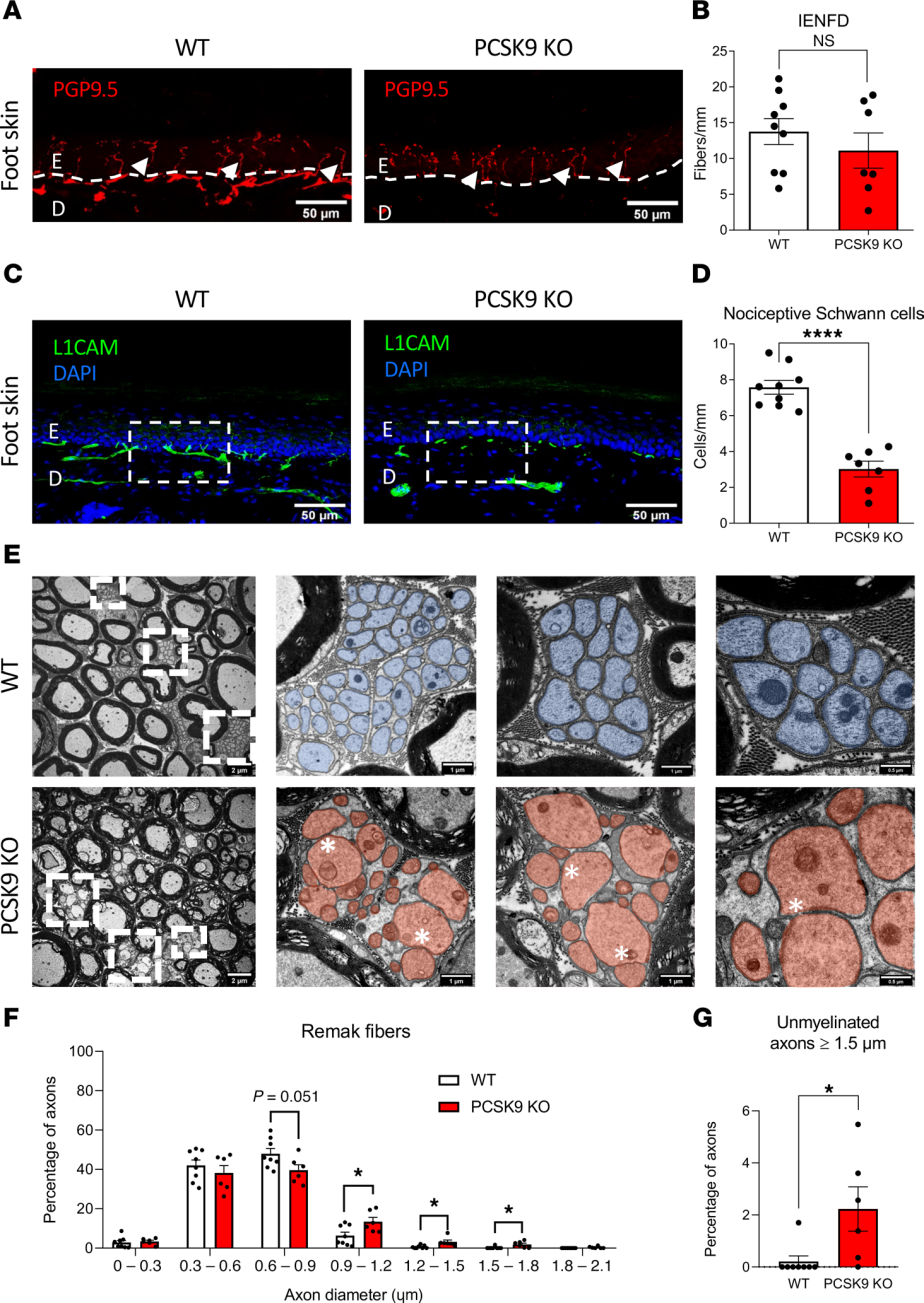
*PCSK9* deficiency is associated with loss of nociceptive Schwann cells and C-fiber axonal swelling. (**A**) Representative image of intraepidermal nerve fiber density (IENFD) (arrowheads above the dotted line delineating epidermis [E] and dermis [D]) in the foot skin with an immunostaining against *PGP9.5* (red). Scale bars: 50 μm. (**B**) Quantification of IENFD in WT (*n* = 9) and *PCSK9-*KO (*n* = 7). (**C**) Representative image of nociceptive Schwann cells in the foot skin with an immunostaining against *L1CAM* (green). Scale bars: 50 μm. (**D**) Quantification of numbers of nociceptive Schwann cells between WT (*n* = 9) and *PCSK9-*KO (*n* = 7) mice. (**E**) Representative photomicrographs of Remak bundle structure by transmission electron microscopy in WT (small C-fibers are highlighted in blue) and *PCSK9-*KO mice (small C-fibers are highlighted in pink); asterisk indicates axon-axon contacts. Scale bars: main image (2 μm); inset, (1 μm); last inset (0.5 μm). (**F**) Distribution of axon diameter in Remak bundles between WT (*n* = 8) and *PCSK9-*KO (*n* = 6) mice. (**G**) Percentage of small unmyelinated C-fibers with a diameter greater than 1.5 μm in WT (*n* = 8, ≥ 2035 axons were analyzed) versus *PCSK9-*KO mice (*n* = 6, ≥ 1727 axons were analyzed). Data are represented as mean ± SEM and analyzed by unpaired *t* test (**B**, **D**, and **G**) and multiple *t* test (**F**). *P < 0.05 and ****P < 0.0001.

Given that pain sensation is transmitted by small nerve fibers innervating the epidermis, we assessed intraepidermal nerve fiber density (IENFD) in the foot skin of WT and *PCSK9-*KO mice using *PGP9.5* as a marker of terminal nerve fibers. Although we found similar IENFD in control and *PCSK9-*KO mice at 10 weeks (8.5 ± 1.5 and 5.9 ± 0.9 fibers/mm, respectively; *P* = 0.1914) (Supplemental Figure 3A) and 24 weeks (13.7 ± 1.8 and 11.1 ± 2.4 fibers/mm respectively; *P* = 0.1952*)* (Figure 3, A and B), we observed a significant decrease in the number of specialized SC called nociceptive SC (nSC) (39) in the foot skin of *PCSK9-*KO mice compared with WT, at 10 weeks (3.3 ± 1.1 and 7.5 ± 0.3 cells/mm, respectively, *P* = 0.0046) (Supplemental Figure 3B) and 24 weeks (3.0 ± 1.4 and 7.5 ± 0.3 cells/mm, respectively; *P* < 0.0001) (Figure 3, C and D) by immunostaining for *L1CAM*, a protein highly expressed in this cell type (40). This decrease was further confirmed in 24-week-old mice with the quantification of nSC expressing *PGP9.5*/DAPI (Supplemental Figure 3, C–E). Thus, although *PCSK9*-KO mice do not exhibit any changes in terminal nerve fiber density within the skin, the number of nSCs is significantly reduced, indicating that *PCSK9* deficiency alters the nociceptive glio-neural complex.

### PCSK9 deficiency alters the morphology and the conduction velocity of peripheral nerve fibers.

**Figure 4:**
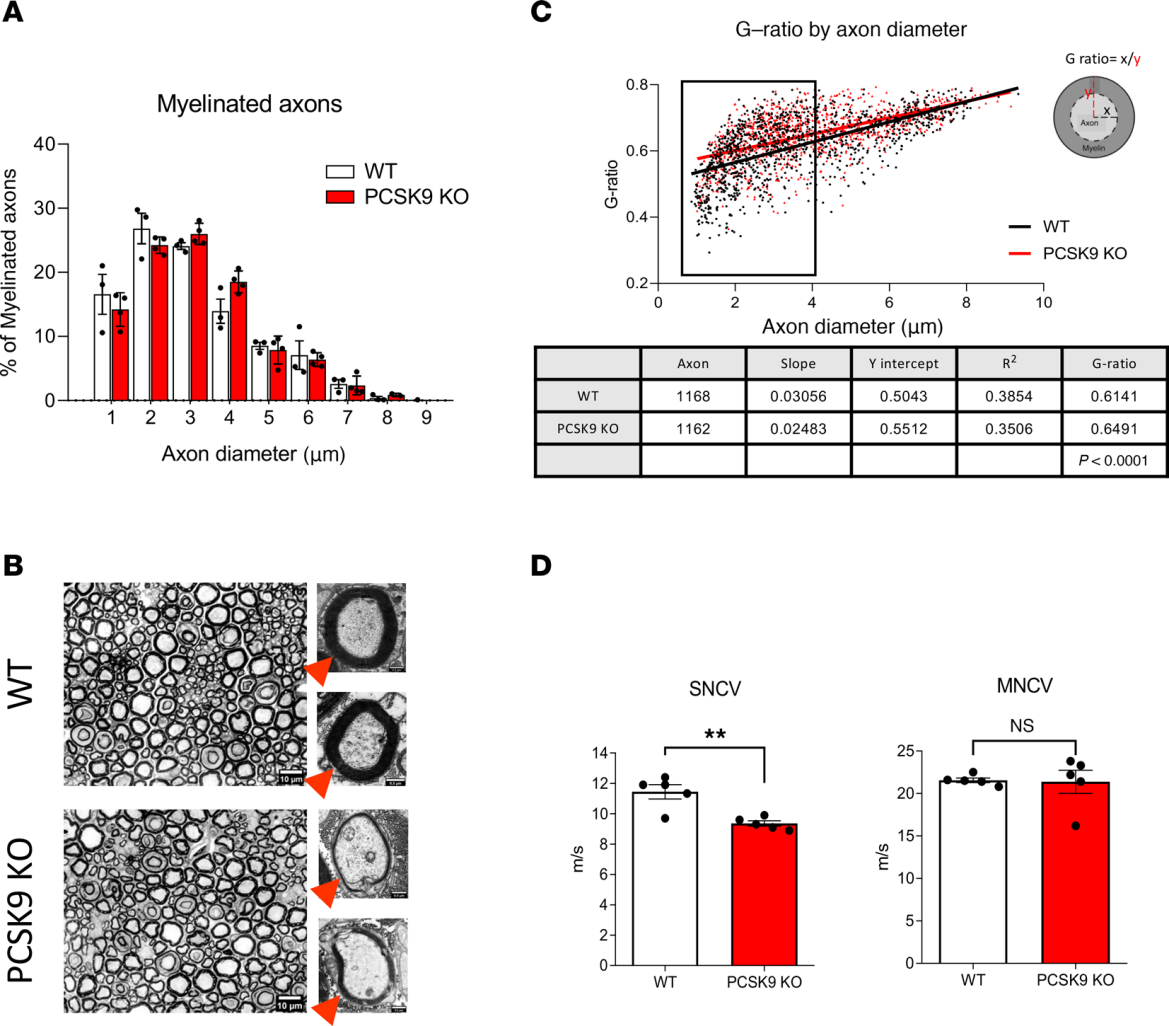
*PCSK9* deficiency is associated with structural and physiological changes in myelinated Aδ-fibers. (**A**) Proportions of myelinated axons distributed according to axon diameter in both WT (*n* = 3) and *PCSK9-*KO (*n* = 4) mice. (**B**) Representative images of toluidine blue staining of myelin in WT and *PCSK9-*KO mice (red arrowheads indicates myelin). Scale bars: main images (10 μm); insets (0.5 μm). (**C**) G-ratio analysis with a summary table in WT (*n* = 4; ≥1,168 axons were analyzed) versus *PCSK9-*KO mice (*n* = 3; ≥1,162 axons were analyzed). (**D**) Sensory nerve conduction velocity (SNCV) and motor nerve conduction velocity (MNCV) compared between WT (*n* = 5) and *PCSK9-*KO (*n* = 5) mice. Data are represented as mean ± SEM and statistically analyzed by multiple *t* test (**A** and **C**) and unpaired *t* test (**D**). **P < 0.01.

We next performed electron microscopy analyses of sciatic nerve sections from these animals and observed axonal swelling of small C-fibers at the level of Remak bundles in *PCSK9-*KO mice (Figure 3E). Quantitative analyses of Remak bundles revealed a shift in the proportion of these C-fibers toward an increase in axon diameter (Figure 3F). This was evidenced by a significant increase in axon diameter in *PCSK9-*KO mice with 2.5% ± 2% of their axons having a diameter above 1.5 mm, compared with only 0.2% ± 0.2% for WT mice (*P* = 0.0221) (Figure 3G). Similar morphological differences between these mice were also observed in their sural nerves that mostly contain nonmyelinated axons (data not shown). Remak bundles normally consist in a single nonmyelinating SC that ensheathes several small C-type axon fibers separately (41). In *PCSK9-*KO mice, most axons within Remak bundles appeared to fasciculate together (Figure 3E), with an abnormal presence of vacuoles within these bundles (Supplemental Figure 3F). We also comparatively assessed the number and morphology of myelinated axons in sciatic nerve sections from WT and *PCSK9-*KO mice. We did not observe any significant difference in the number nor in the diameter of myelinated small-diameter axons (1–5 mm) and myelinated large diameter axons (≥5 mm) between WT and *PCSK9-*KO mice (Figure 4A). Our analysis of the myelination status of sciatic nerve fibers revealed that small, myelinated Aδ-fibers (≤ 5 mm) of *PCSK9-*KO mice had a significant increase in their G-ratio, reflecting a decrease in myelin thickness, compared with those of WT mice (Figure 4, B and C). These morphological differences were associated at the functional level with a significantly reduced sensory nerve conduction velocity (9.36 ± 0.39 vs 11.45 ± 1.04 m/s, *P* = 0.0031) but similar motor nerve conduction velocity (21.38 ± 3.04 vs 21.56 ± 0.61 m/s, *P* = 0.9), in *PCSK9-*KO mice compared with WT, respectively (Figure 4D). Thus, while peripheral nerves of *PCSK9-*KO mice contain an adequate number of axons, they exhibit alterations in peripheral nerve morphology and conduction properties, notably axonal swelling of C-fibers within Remak bundles, a decrease in myelin thickness of small Aδ-fibers, and a corresponding reduction in sensory nerve conduction velocity.

### PCSK9 modulates CD36 but not LDLR expression in peripheral nerves.

**Figure 5:**
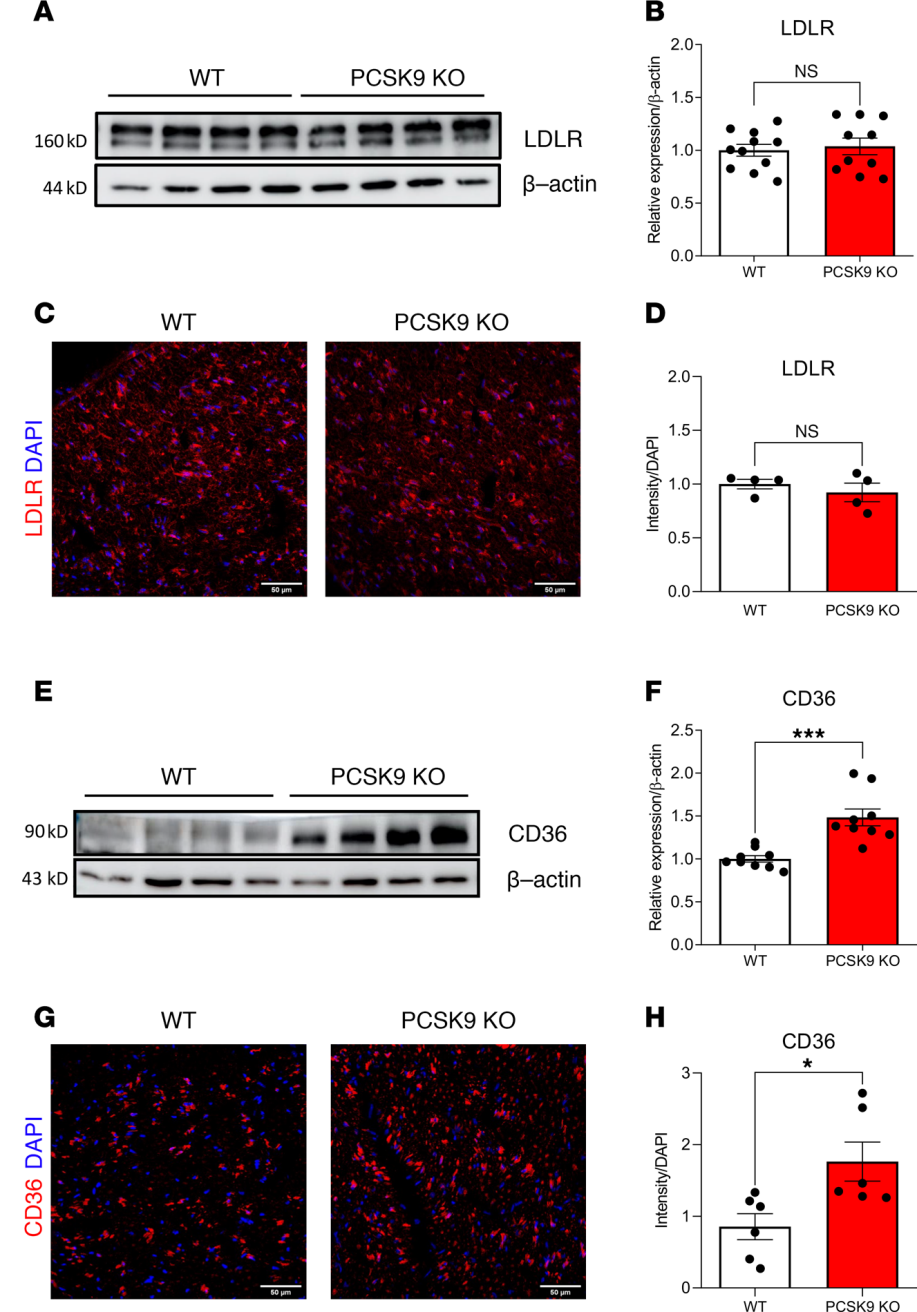
*PCSK9* deficiency results in increased *CD36* expression in peripheral nerves. (**A**) *LDLR* expression in the sciatic nerve by immunoblotting. (**B**) Quantification of *LDLR* expression in 24-week-old WT (*n* = 11) and *PCSK9-*KO mice (*n* = 10). (**C**) IHC for *LDLR* in sciatic nerve sections of 24-week-old WT and *PCSK9-*KO mice. Scale bars: 50 μm. (**D**) IHC quantification of *LDLR* expression in 24-week-old WT (*n* = 4) and *PCSK9-*KO (*n* = 4) nerve sections. (**E**) *CD36* expression in the sciatic nerve by immunoblotting. (**F**) Quantification of *CD36* expression in 24-week-old WT (*n* = 9) and *PCSK9-*KO (*n* = 9). (**G**) IHC for *CD36* in sciatic nerve sections of 24-week-old WT and *PCSK9-*KO mice. Scale bars: 50 μm. (**H**) IHC quantification of *CD36* expression in 24-week-old WT (*n* = 6) and *PCSK9-*KO (*n* = 6) nerve sections. Data are represented as mean ± SEM and statistically analyzed by unpaired *t* test. *P < 0.05 and ***P < 0.001.

To gain insights into the molecular mechanisms underlying the sensory and morphological phenotypes observed in *PCSK9-*KO mice, we assessed the expression of the main *PCSK9* targets in the sciatic nerves of these animals. Surprisingly, we did not detect any change in *LDLR* levels in the peripheral nerves of *PCSK9-*KO mice compared with WT (Figure 5, A–D), in contrast to what is observed in the liver (Supplemental Figure 4, A and B). Neutral results were also obtained when analyzing the expression of *LRP1*, *VLDLR*, and *apoER2* receptors (Supplemental Figure 5, A–C). In contrast, we observed a significant increase in the expression of *CD36* in sciatic nerves of *PCSK9-*KO compared with WT mice (Figure 5, E–H), as in the liver (Supplemental Figure 4, A and C). We established that upregulation of *CD36* in the peripheral sciatic nerve of *PCSK9-*KO mice was prominent in nerve fibers as well as in myelinating and nonmyelinating SC (Supplemental Figure 6, A and B). Of note, in the DRG, *CD36* was expressed at extremely low levels, and its expression, as well as that of the *LDLR*, was not significantly altered in *PCSK9-*KO mice (Supplemental Figure 7, A–D). These findings demonstrate that the absence of *PCSK9* specifically induces the upregulation of *CD36* but not *LDLR* or other target receptors in peripheral nerves.

### PCSK9 deficiency is associated with lipid accumulation in peripheral nerves.

**Figure 6:**
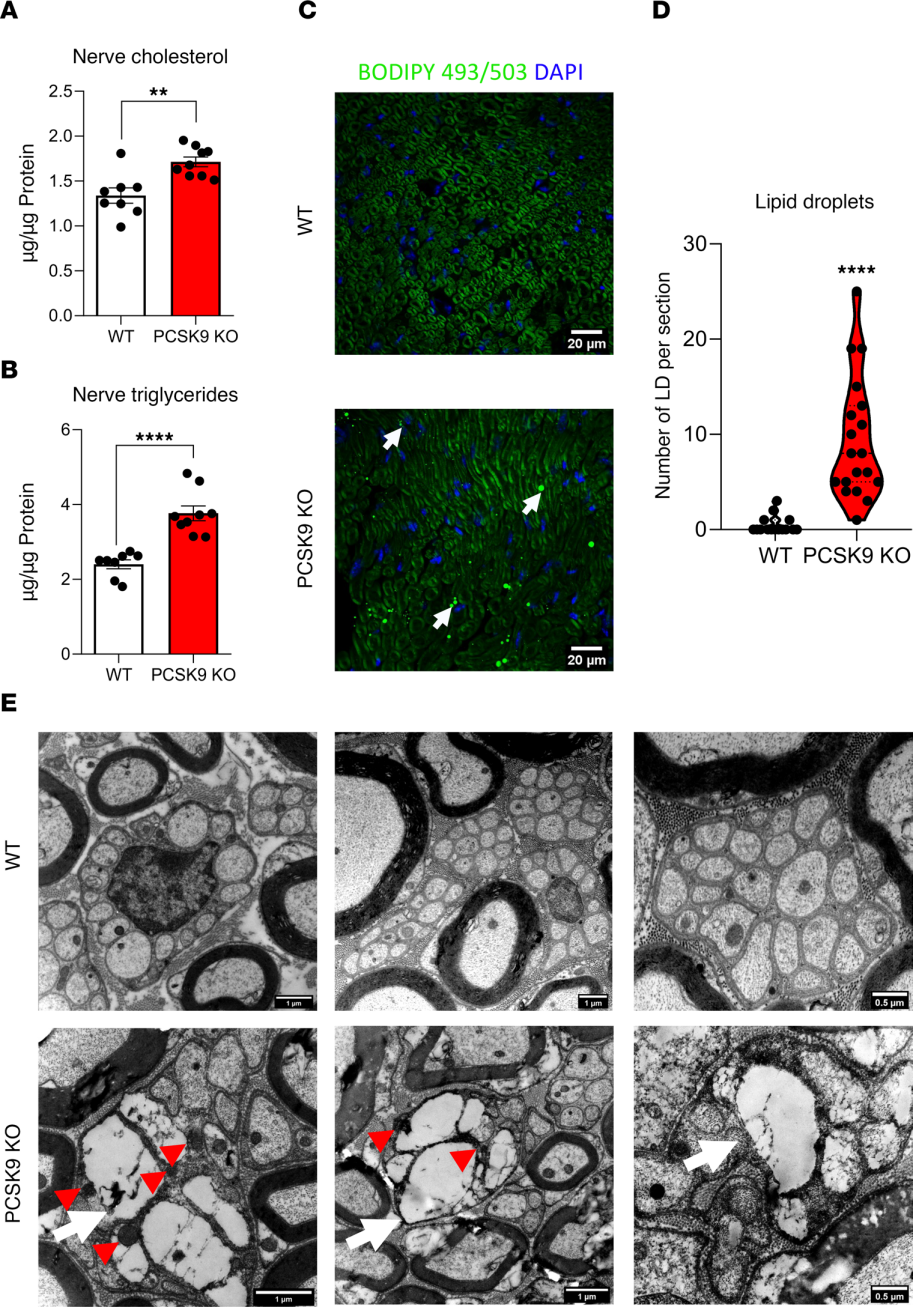
*PCSK9* deficiency results in increased lipid concentration in peripheral nerves. (**A**) Cholesterol levels in the sciatic nerve of 24-week-old WT (*n* = 8) and *PCSK9-*KO (*n* = 9) mice. (**B**) Triglyceride levels in the sciatic nerve of 24-week-old WT (*n* = 8) and *PCSK9-*KO (*n* = 9) mice. (**C**) Visualization of lipid droplets in sciatic nerve sections using BODIPY 493/503 staining (arrows: lipid droplets). Scale bars: 20 μm. (**D**) Quantification of the number of lipid droplets per section from 24-week-old WT (*n* = 3) and *PCSK9-*KO (*n* = 4) mice. (**E**) Representative photomicrographs of sciatic nerve sections by transmission electron microscopy in WT and *PCSK9*-KO mice are shown. Scale bars: 1 μm; final image (0.5 μm). White arrows indicate lipid droplets; red arrowheads show the associated mitochondria. Data are represented as mean ± SEM and statistically analyzed by unpaired *t* test. **P < 0.01 and ****P <0.0001.

We next evaluated whether the upregulation of the fatty acid transporter *CD36* in peripheral nerves altered their lipid content. We measured cholesterol and triglyceride concentrations in sciatic nerve extracts and observed respective increases of 30% and 50% in *PCSK9*-KO compared with WT mice (Figure 6, A and B). Liver lipid contents were also measured and were consistent with the results of previous studies (42) (Supplemental Figure 4, D–F). In contrast, we did not observe any change in cholesterol and triglyceride concentrations in DRG extracts from control and *PCSK9-*KO mice (Supplemental Figure 7, E and F). In peripheral nerves, cholesterol and triglyceride contents correlated strongly with *CD36* protein expression (Supplemental Figure 8, A and B). Furthermore, neutral lipid staining of sciatic nerve sections showed an accumulation of lipid droplets in *PCSK9-*KO mice compared with control animals (Figure 6, C and D). Structurally, electron microscopy revealed that *PCSK9-*KO but not WT peripheral nerves displayed lipid droplets within Remak bundles (Figure 6E). Taken together, these results indicate that *PCSK9* deficiency leads to an abnormal lipid accumulation within peripheral nerves.

### Mitochondrial defects and AC accumulation in peripheral nerves of PCSK9-KO mice.

**Figure 7:**
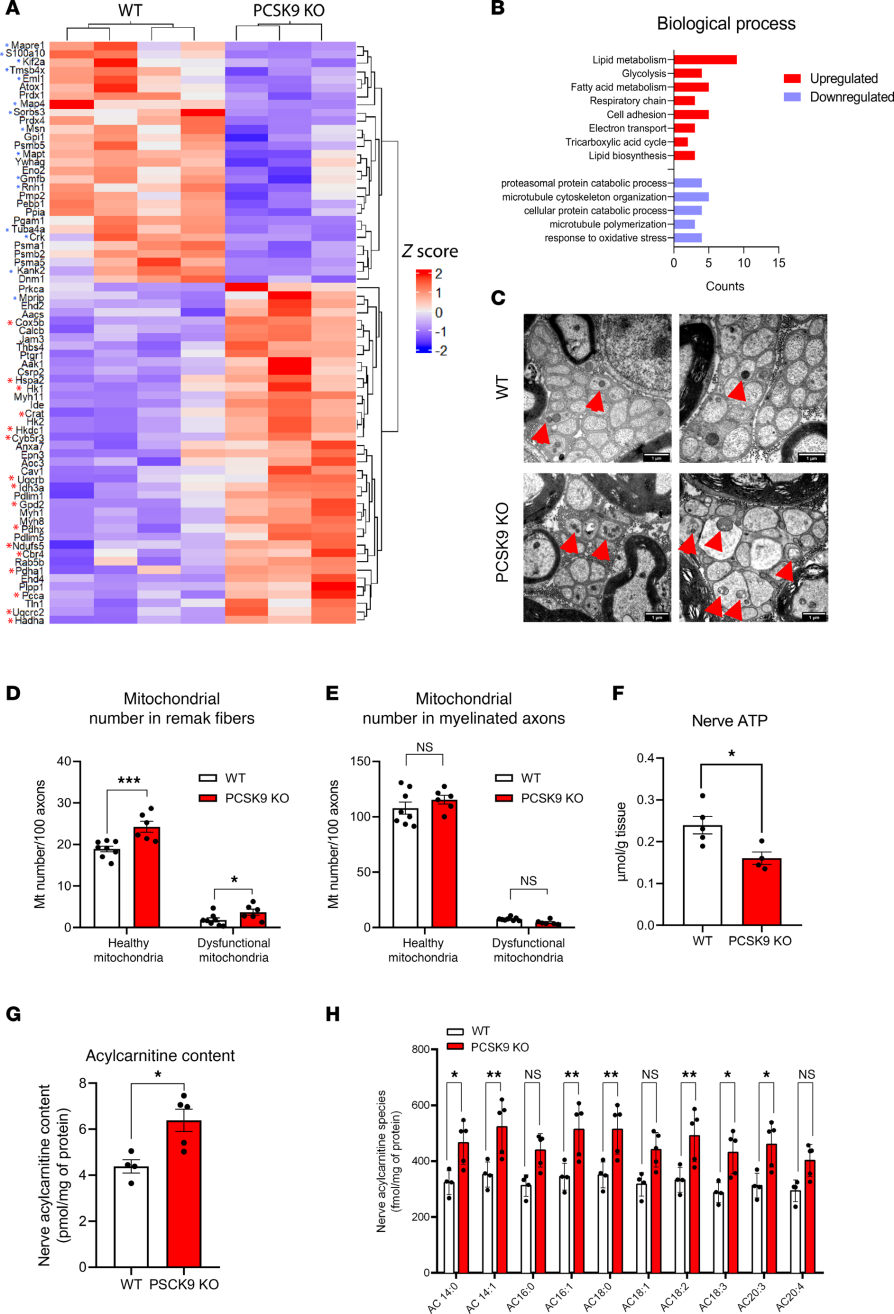
Proteomic profile and mitochondrial abnormalities of *PCSK9-*KO nerves. (**A**) Heatmap showing relative protein expression values (*z* score) of *n* = 62 proteins that are differentially expressed in the nerves of WT (*n* = 4) compared with *PCSK9*-KO (*n* = 3) mice (red asterisk represents upregulated mitochondrial proteins, blue asterisk represents downregulated microtubule proteins). (**B**) Gene ontology annotation of the top upregulated (red) and downregulated (blue) terms in biological processes sorted by *P* value. (**C**) Representative TEM images of Remak bundles (red arrows: healthy mitochondria; red arrowheads: abnormal mitochondria). Scale bars: 1 μm. (**D**) Quantification of total mitochondrial numbers in C-fibers with the ratio of abnormal mitochondria per 100 axons in WT (*n* = 8; ≥2,035 axons were analyzed) and *PCSK9*-KO mice (*n* = 6; ≥1,727 axons were analyzed). (**E**) Quantification of total mitochondrial numbers in myelinated Aδ-fibers with the ratio of abnormal mitochondria per 100 axons in WT (*n* = 8; ≥479 axons were analyzed) and *PCSK9-*KO (*n* = 6; ≥446 axons were analyzed). (**F**) ATP levels in the sciatic nerves of WT (*n* = 5) and *PCSK9-*KO (*n* = 4) mice. (**G**) Quantification of total AC in the sciatic nerve of 24-week-old WT (*n* = 4) and *PCSK9-*KO (*n* = 5) mice. (**H**) Increase of different AC molecular species in the sciatic nerve of 24-week-old WT (*n* = 4) and *PCSK9-*KO (*n* = 5) mice. Data are represented as mean ± SEM and statistically analyzed by unpaired *t* test. *P *<* 0.05, **P < 0.01, ***P < 0.001.

To further unravel the pathophysiological onset of the sensory phenotype of *PCSK9-*KO mice, we next performed an untargeted proteomic investigation of their sciatic nerves. *PCSK9-*KO mice showed a significant upregulation of several proteins involved in mitochondrial lipid metabolism and a downregulation of cytoskeletal proteins compared with WT mice (Figure 7A). Major upregulated proteins are implicated in β-oxidation such as carnitine O-acetyl transferase (*Crat*) and Trifunctional enzyme subunit α (*Hadha*), enzymes of the TCA cycle including isocitrate dehydrogenase [NAD] subunit α (*Idh3a*), and proteins of the electron transport chain including complex I (*Ndufs5*), complex III (*Uqcrc2*, *Uqcrb*), and complex IV (*Cox5b*). Interestingly, Caveolin-1, a protein necessary for the localization and endocytosis of *CD36* at the membrane (43), was upregulated by 50% in *PCSK9-*KO sciatic nerves, mirroring a similar increase of magnitude in *CD36* expression. The proteomic analysis also revealed a downregulation of proteins involved in proteasomal catabolic processes, such as *Psma1*, *Psmb2*, and *Psma5*, as well as microtubule organization proteins including, *Map4*, *Mapt*, *Msn*, *Sorbs3*, and the peripheral myelin protein 2 (*PMP2*), a constituent of peripheral nerve myelin (Figure 7A). Biological process enrichment analysis of *PCSK9-*KO nerve proteome indicated increased overall processes of lipid metabolism and electron transport chain processes and downregulation of processes involved in microtubule cytoskeleton organization and the capacity to respond to oxidative stress (Figure 7B), with most of the identified upregulated proteins clustered as mitochondrial components (Supplemental Figure 8C). To corroborate these results, we analyzed mitochondria number and morphology in sections of sciatic nerve by electron microscopy. We observed an increase in both the number of mitochondria and the proportion of abnormal (swollen, onion-like, herniated, vacuolated) mitochondria (Supplemental Figure 8D) in C-fibers of *PCSK9-*KO mice (Figure 7, C and D), indicative of enhanced mitophagic processes. This was not seen in myelinated Aδ-fibers (Figure 7E). In line with the presence of defective mitochondria, a significant reduction in sciatic nerve ATP production was observed in *PCSK9-*KO mice compared with WT mice (Figure 7F). As there was an overload of lipids and an upregulation of several proteins involved in mitochondrial β-oxidation in *PCSK9-*KO peripheral nerves, we quantified the level of AC, a toxic intermediate of mitochondrial fatty acid β-oxidation (44). Interestingly, we found a significant increase of AC in the peripheral nerves of *PCSK9-*KO mice compared with those of control mice (Figure 7, G and H). Of note, high levels of AC have been associated with the development of peripheral neuropathy in humans and mice (44, 45). These combined observations indicate that the accumulation of toxic lipid intermediates associated with an alteration of mitochondria in *PCSK9-*KO mice likely contribute to their neuropathic phenotype.

## Discussion

In this study, we found that *PCSK9* gene inactivation in male mice induces a neuropathic phenotype characterized by a reduction in thermal and mechanical pain sensations. These sensory deficits were associated with a reduction in sensory nerve conduction properties, despite no significant alteration in the number of sensory axons in the nerve or in the density of their terminal endings in the skin. In contrast, we observed a decrease in myelin thickness of small-diameter A-myelinated fibers and a reduction in the number of terminal nSC at the border between the dermis and the epidermis. We also established that *PCSK9* deletion induces an upregulation of the fatty acid transporter *CD36* associated with an abnormal accumulation of lipids in peripheral nerves. We showed that *PCSK9* deficiency leads to an increase of AC concentration and mitochondrial defects. In light of these observations, we propose a model in which *PCSK9* finely tunes the internalization of lipids in peripheral nerves through the regulation of *CD36* receptor.

We notably show that *PCSK9* is physiologically expressed in the PNS at the level of the sensory neurons and in both myelinating and nonmyelinating SC, which had initially been suggested by the detection of *PCSK9* mRNA in a rat SC line (7) and in human tibial nerves (11). We also observed that mice lacking *PCSK9* exhibit sensory defects, characterized by a decrease in their ability to perceive light mechanical stimuli and an increase in thermal pain sensitivity at 10 weeks of age and, subsequently, a reduced sensitivity to acute mechanical and thermal pain stimuli at 24 weeks.

In line with their neuropathic phenotype, we observed structural alterations at the level of terminal endings of peripheral nerves in the skin of *PCSK9*-KO mice. Intriguingly, while we did not observe any significant decrease in IENFD in their skin at the latest age of our analyses, we noticed a significant reduction in the number of nSCs, a nonneuronal cutaneous cell subtype closely connected with sensory endings and known to play a critical role in tactile sensations (39, 46). Consistent with these studies, the reduction in the number of nSC in *PCSK9-*KO mice was associated with a significant decrease in responses to mechanical pain stimuli at both 10 and 24 weeks of age. Intriguingly, a study from Rinwa et al., reported a decrease in IENFD and the development of hyperalgesia after ablation of nSC (47). A potential difference that could explain this apparent discrepancy is that, by targeting *Sox10*-expressing nSC, Rinwa et al. probably affected the functionality of some nonmyelinated as well as myelinated SCs that also express *Sox10* in the skin, which in turn might have induced neuroinflammation, contributing to the hyperalgesia phenotype. Their findings also contrast with earlier results showing that silencing nSC led to a significant increase in mechanical pain threshold rather than hyperalgesia (39).

At the level of the sciatic nerves, we observed axonal swelling of unmyelinated C-fibers in Remak bundles, as well as a reduction in the thickness of the myelin sheath around small-diameter A-myelinated axons in *PCSK9-*KO mice. These 2 types of fibers, referred to as unmyelinated C-fibers and myelinated Aδ-fibers, respectively, are specifically involved in the transmission of mechanical pain (48, 49). Axonal swelling of unmyelinated C-fiber nociceptors represents an early stage of neurodegeneration (50–53). Studies targeting mitochondrial transcription factor A (*Tfam*) or serine/threonine kinase *LKB1* in SC have thus shown similar axonal swelling and C-fiber degeneration, accompanied by reduced sensitivity to noxious stimuli (54, 55). Additionally, targeting *Rheb* (Ras homolog enriched in brain) expression in SCs has also resulted in a significant number of swollen axons in Remak bundles, indicative of degenerating axons (56). In our analysis, we observed C-fiber axonal swelling coupled with a downregulation of proteins involved in proteasomal catabolic processes and microtubule biology, both indicators of neurodegenerative processes (57, 58). This suggests that C-fibers in *PCSK9-*KO mice are undergoing degeneration, likely contributes to mechanical and thermal pain stimuli. However, we did not observe any significant change in Aδ-fiber axonal diameter at the latest stage of our analysis. This aligns with previous studies (54, 55) and supports the notion that C-fibers, which have higher energy demands compared with Aδ-fibers (59), are more dependent on metabolic support.

We also showed that *PCSK9-*KO mice present with an increased expression of *CD36* in peripheral nerves, whereas the expression of other known *PCSK9* targets (*LDLR*, *VLDLR*, *ApoER2*, and *LRP1*) were unaltered in these tissues compared with WT mice. The absence of modulation of *LDLR* abundance in *PCSK9-*KO mouse peripheral nerves is surprising, given the overt role that *PCSK9* plays in *LDLR* intracellular degradation in hepatocytes and many other cell types (60, 61). Nevertheless, the lack of *LDLR* modulation in neuronal tissues such as the brain and cerebellum of *PCSK9-*KO mice, has previously been reported by others (62–64). Although the mechanism is still unclear, several proteins have been proposed as intermediates to either enhance or impede *PCSK9*-mediated degradation of the *LDLR*. Thus, Annexin 2 (65, 66) and glypican-3 (67) were shown to inhibit *PCSK9* binding to *LDLR*, whereas cyclase-associated protein 1 (68) enhances its binding capacity. The differential expression of these proteins between the hepatic and nervous system could explain the distinct regulatory functions of *PCSK9* on *LDLR* between these tissues. In contrast, an increase in *CD36* expression has been reported in many organs of *PCSK9*-KO mice, including the liver, adipose tissue, heart, and kidneys (42, 69–71). *CD36* is a scavenger receptor involved in fatty acid and triglycerides metabolism that plays an important role in lipid accumulation (72). The increase in *CD36* expression resulting from *PCSK9* gene deletion was systematically associated with an abnormal accumulation of lipid droplets in other tissues (42, 69–71). Accordingly, we also found an increase in lipid contents in the peripheral nerves of *PCSK9*-KO mice proportional to the levels of *CD36* expression. Peripheral nerves have high energy demands, and circulating fatty acids can be taken up by neurons and SCs to be oxidized in mitochondria for ATP production. However, excessive uptake of fatty acids can lead to the production of toxic lipid intermediates and reactive oxygen species (ROS), which in turn can cause mitochondrial dysfunction and nerve injury (21, 54, 73). Consistent with this hypothesis, we observed increased expression of mitochondrial enzymes involved in β-oxidation, TCA cycle, and electron transport chain in the nerves of *PCSK9*-KO mice. The accumulation of lipids induced by *CD36* overexpression may, therefore, overwhelm the ability of SC and axons to metabolize these compounds effectively, leading to impaired ATP production. Additionally, the presence of structurally abnormal and dysfunctional mitochondria within unmyelinated Remak bundle fibers of *PCSK9-*KO mice was associated with an accumulation of AC, a toxic intermediate of fatty acid β-oxidation. These findings are consistent with reports showing an increased proportion of dense, depolarized mitochondria in peripheral axons of mouse models of diabetic neuropathy (74, 75). Alterations of mitochondrial metabolism and an accumulation of AC were also observed in cardiomyocytes of *PCSK9-*KO mice, in addition to enhanced *CD36* expression and the presence of lipid droplets in the heart of these animals (70). Our observations show that, in the absence of *PCSK9*, peripheral nerves exhibit increased *CD36* expression and an abnormal accumulation of lipids. Given that the mechanism behind the *PCSK9*-induced degradation of *CD36* is still elusive (1), we cannot fully rule out the possibility that *CD36* might not be the central mechanism at the origin of the dysfunctional phenotypes observed in the heart (70), β cells (76), kidneys (71), and peripheral nerves of *PCSK9*-KO mice. Definitive evidence can only be established using *PCSK9*/*CD36*–double KO animals, which is beyond the scope of the present study. Collectively, our observations support a role for *PCSK9* in controlling *CD36* levels in peripheral nerves, thereby protecting nerve cells against excessive lipid uptake and accumulation that can lead to neuronal lipotoxicity.

One apparent limitation to the present study is that our in vivo results contrast with the fact that, over the last decade, only 1 documented case of peripheral neuropathy has been causatively linked to *PCSK9* inhibitors usage (12). Even if long-term clinical trials of *PCSK9* inhibitors have not shown any sign of negative neurocognitive events (4, 77), there was no specific evaluation of sensory-motor endpoints in these studies. Furthermore, these drugs are monoclonal antibodies that specifically antagonize the interaction domain between *PCSK9* and the *LDLR* but not the *PCSK9* region that interacts with *CD36* (1, 71). As a result, *PCSK9* inhibition will not increase *CD36* abundance in peripheral nerves and will not lead to a neuropathic phenotype. These drugs might even prove neuroprotective, as they increase the plasma concentration of *PCSK9* (78) with a preserved ability to inhibit *CD36*. Likewise, these monoclonal antibodies as well as anti-*PCSK9* siRNA inclisiran only target liver-derived circulating *PCSK9* (6, 79). Given that *PCSK9-*KO mice totally lack *PCSK9*, the neuropathic phenotype observed in these mice could result either from the absence of *PCSK9* expression locally in SC and sensory neurons or from the systemic lack of *PCSK9* in the circulation. In this regard, it will be informative to test these possibilities in mice specifically lacking *PCSK9* in peripheral nerve cells (SC, sensory neurons) or in the liver. A second limitation of our study is that a neuropathic phenotype was only found significant in *PCSK9-*KO male mice, adding to the long list of sex-specific and tissue-specific phenotypes previously reported in mice lacking *PCSK9* (80, 81). Finally, our analyses were performed at 2 time points only (10 and 24 weeks of age), and we ignore whether the neuropathic phenotype of *PCSK9-*KO male mice will continue to worsen over time.

Nevertheless, our findings significantly advance our understanding of peripheral nerve health and function that rely on an accurate equilibrium between lipid synthesis, circulating lipid uptake, β-oxidation, and de novo lipogenesis. The discovery of a role for *PCSK9* in peripheral nerve lipid homeostasis through *CD36* regulation opens avenues of research to elucidate how peripheral nerves deal with fluctuating levels of circulating lipids for their important energy and structural needs while avoiding lipotoxicity. Our findings are particularly relevant in the context of diabetic neuropathy, as dyslipidemia is increasingly recognized as an important risk factor for this condition (24, 29, 82). Our findings also provide insights to better understand certain cases of acquired demyelinating disorders of peripheral nerves (e.g., Guillain-Barré syndrome, anti–myelin-associated glycoprotein neuropathy) as well as nerve injury and remyelination, where lipids are considered limiting resources (83, 84). Future investigations are needed to understand the implication of *PCSK9* and target receptors in order to design targeted therapeutic approaches for these pathologies.

## Methods

### Sex as a biological variable

We initially used both male and female animals in our behavioral analyses. Since *PCSK9-*KO male mice showed a marked sensory phenotype, we explored this further by performing all the subsequent experiments in males only.

### Mice

*PCSK9-*KO mice (B6; 129S6-Pcsk9tm1Jdh/J) were purchased from the Jackson Laboratory and backcrossed for 10 generations to C57BL/6J mice. *PCSK9* heterozygous mutant (+/–) mice from the F10 generation were intercrossed to generate littermate controls (+/+) and *PCSK9-*KO (–/–) animals. Littermate controls (referred to as WT mice throughout) and *PCSK9-*KO mice were subsequently used in all experiments. Mice were kept under a controlled light/dark cycle (12 hours of light/12 hours of dark), under temperature-controlled conditions (21°C) and standard humidity. Mice were fed chow diet ad libitum with free access to water. Mice were anesthetized with isoflurane and euthanized by intracardiac puncture. Blood samples were collected for plasma isolation by centrifugation (2,000*g* at 4°C for 15 minutes), and tissues (liver, DRG, sciatic nerves, foot skin) were snap frozen in liquid nitrogen. Plasma and tissue samples were stored at –80°C until use. All biochemistry and anatomy analyses were performed on 24-week-old mice unless mentioned otherwise.

### IHC

Right after euthanasia, mice were perfused with 20 mL 1× PBS followed by 15 mL of 4% paraformaldehyde (PFA) dissolved in 1× PBS. Sciatic nerves, foot skin, and DRGs were dissected and fixed overnight in 4% PFA at 4°C. Tissues samples were washed 3 times (10 minutes) in cold PBS, cryoprotected overnight in a PBS solution containing 30% sucrose, embedded in OCT compound, and stored at –80°C until use. Frozen tissues were cut into 14 μm sections (20 μm for foot skin), placed onto glass slides, and dried on a heating plate for 10 minutes. The slides were stored at –20°C until use. Sections were rehydrated in PBS, permeabilized with PBT (PBS + 0.1% Triton X-100), and blocked in donkey serum (Sigma-Aldrich) prior to an overnight incubation with primary antibodies (Supplemental Table 1). For *PCSK9* immunostaining, we added an antigen retrieval step by incubating the sections for 10 minutes in 10 mM citrate buffer (pH 6.0) at 95°C. Slides were washed 3 times (10 minutes) in PBT, incubated with fluorescent secondary antibodies (Supplemental Table 1) for 1 hour, and washed again as above. Nuclei were counterstained with DAPI (0.5 μg/mL), slides were washed again and then mounted and kept at 4°C.

### RNAscope

RNA in situ hybridization was performed using the RNAscope Multiplex Fluorescent Reagent Kit Assay (Advanced Cell Diagnostics Inc.) according to the manufacturer’s instructions. RNAscope probes used were as follows: *Pcsk9* (catalog 498111), *Sox10* (catalog 435931), *Rbfox3* (*NeuN*; catalog 313311-C2), and *Ldlr* (catalog 443701). Nuclei were ultimately counterstained with DAPI.

### Image acquisition and analysis

Images were acquired on a laser-scanning confocal microscope Eclipse Ti2 (Nikon) with identical parameters (laser power, iris diameter, and gain) to acquire images from all sections.

### Mouse and human primary SC culture

Primary SC cultures were isolated as follows. Sciatic nerves of 6 mouse pups between P2 and P4 were collected in L15 medium (Thermo Fisher Scientific) and incubated for 30 minutes in 2 mg/mL collagenase II and then 10 minutes in 0.25 % trypsin containing EDTA at 37°C. Nerves were dissociated into single-cell suspensions with a 1 mL pipette and then through an 18 and 21 G needle. SCs were resuspended in primary SC medium (ScienCell, 1701) supplemented with 1 μM insulin, 2 μM forskolin, and 10ng/mL Nrg1β (85).

Human primary SCs isolated from human spinal nerve were commercially obtained from Sciencell (catalog 1700). Cells were maintained in primary SC medium.

### Mouse primary sensory neurons culture

Primary sensory neuron cultures were isolated as follows. DRG of 6 mouse pups between P2 and P4 were collected in PBS-glucose (0.3 μM) and incubated for 30 minutes in 0.125% collagenase D (Roche) and then 10 minutes in 0.25 % trypsin at 37°C. Ganglia were dissociated into single-cell suspensions with a glass Pasteur pipette. Sensory neurons cells were resuspended in Neurobasal (NB) (Thermo Fisher Scientific, 21103049) supplemented with B27 (Thermo Fisher Scientific, 17504044).

### Biochemistry and Western blot analyses

Cholesterol and triglyceride content in plasma and tissue samples was determined using colorimetric assays (Diasys). Concentration of *PCSK9* in the plasma and tissue samples was assessed by ELISA (Quantikine MPC900, R&D Systems). ATP concentrations were determined from fresh sciatic nerves using ATP Assay Kit (Abcam, ab83355) following the manufacturer’s instructions. Before measuring the colorimetric signals at 570 nm, samples were deproteinized with a Deproteinizing Sample Preparation Kit (Abcam, ab204708). Protein extracts were obtained after lysis of tissues in 350-μL Tris HCl buffer (pH 9.5; 10 mM) containing EDTA (1 mM), NaCl (150 mM) supplemented with the Halt Protease, and Phosphatase Inhibitor Cocktail (Thermo Fisher Scientific). Proteins (approximately 20 μg) were separated by sodium dodecyl sulfate 7.5% polyacrylamide gel electrophoresis and transferred onto a nitrocellulose membrane. Membranes were blocked in tris-buffer saline containing 5% (w/v) nonfat dry milk, 0.1% (v/v) Tween 20 (TBS) for 1 hour at RT before incubation with the primary antibodies (Supplemental Table 1) overnight at 4°C. Membranes were washed in TBS-T and incubated for 90 minutes at 25°C with HRP-conjugated secondary antibodies (Supplemental Table 1). Immunoreactive bands were detected by chemiluminescence (Amersham ECL Prime Western Blotting Detection Reagent, GE Healthcare Life Sciences), and images were acquired with an Amersham Imager 600 (GE Healthcare) and analyzed with the ImageJ software (NIH). Throughout, results were normalized to β-actin expression.

### Behavioral assessment of peripheral neuropathy

#### von Frey test.

Mice were placed in a plastic chamber (10 × 10 × 14 cm) on an elevated wire grid. They were habituated to the testing apparatus for 1 hour/day during a week before behavioral testing. The plantar surface (glabrous) of the hind paw was stimulated with a set of calibrated von Frey filaments (0.008–6 g) (Bioseb; In Vivo Research Instruments). The PWT was determined as described previously (86). von Frey responses were determined as the percentage of withdrawal using specific filament (0.07, 0.16, 0.4, 0.6, 1,1.4 g). Each filament was tested 5 times in increasing order.

#### Pinprick test.

Mice were placed in a plastic chamber on an elevated wire grid. The plantar surface of the hind paw was stimulated with a pin gently applied without moving the paw or penetrating the skin. Pin stimulation was repeated 5 times on different areas with a 1- to 2-minute interval between each stimulation. The percentage of positive attempts (paw withdrawal) was calculated.

#### Hargreaves test.

In the Hargreaves test, we measured the response to radiant heat. Mice were placed in a plastic chamber on a glass floor, and the infrared heat stimulus from the plantar test (Ugo Basile) was applied to the hind paw. The latency for the animal to withdraw the hind paw was measured. The beam intensity was adjusted at 20% so that control mice displayed a latency of 8–12 seconds. A cutoff time of 30 seconds was used to avoid tissue damage.

#### Brush test.

To assess sensitivity to dynamic light touch, we used a brush test. The plantar surface of the hind paw was stimulated with a soft paintbrush by gently stroking from heel to toe. The brush stimulation was repeated 5 times with an interval of 1 minute and the percentage of paw withdrawals from 5 stimuli was calculated.

#### Rotarod test.

Gross motor ability and coordination were assessed using a rotarod apparatus (Bioseb). Mice were first placed on the apparatus for 30 seconds with no rotation and thereafter for 2 minutes at a constant low-speed rotation (4 rpm). Those that fell from the rod at 4 rpm were placed again on it until they were able to stay for 1 minute. Mice were tested in accelerating conditions from 4 to 40 rpm over 2-minute period. The time during which the animal walked on the rod before falling was collected (maximum value, 120 seconds). The occurrence of 2 consecutive passive rotations — i.e., without walking but accompanying the rod — was considered as a fall. Each mouse was tested 3 times per day with intertrial intervals of 5 minutes.

#### Inverted grip test.

Grip strength was measured by testing the ability of the mouse to remain clinging to an inverted wire grid for a maximum period of 1 minute.

### Nerve conduction studies (NCV)

Mice (24 weeks old) were anesthetized with isoflurane at doses of 4%–5% for induction and 1%–2% for maintenance. The core temperature was maintained at 34°C with a heating pad. Stainless steel needle electrodes (Natus Biomedical) were cleaned with 70% alcohol between animals. Using the Natus UltraPro S100 EMG/NCS system and the Keypoint 4 software, sensory NCV was determined by dorsal paw recordings and stimulating electrodes on the ankle. For sensory NCV, the latency of onset (milliseconds) of the sensory nerve action potential after supramaximal antidromic stimulation of the sural nerve at the ankle was divided by the distance between the recording and stimulation electrodes (measured in millimeters using a Vernier caliper). Motor NCV was calculated by subtracting the distal from the proximal latency (measured in milliseconds) from the stimulus artifact of the take-off of the evoked potential, and the difference was divided by the distance between the 2 stimulating electrodes (measured in millimeters using a Vernier caliper).

### IENFD and nSC quantification

IENFD quantification was performed on WT and *PCSK9-*KO mice. Both right and left plantar surface of the hind paw were collected and fixed overnight with 4% PFA. Tissues were rinsed with 1× PBS and then incubated in 30% sucrose PBS solution overnight. Tissues were cryoembedded in mounting media (OCT) and sectioned at 20 μm thickness before being processed for IHC with an antibody against *PGP9.5* and *L1CAM* (Supplemental Table 1). Fluorescence images were collected by confocal microscopy. Approximately 10 images per stack were flattened using the ImageJ software. Six different skin sections were measured for each foot. The number of intraepidermal nerve fibers and nSCs (*PGP9.5*^+^/DAPI^+^) or (*L1CAM*^+^/DAPI^+^) were presented as the mean number of fibers and cells per linear millimeter of epidermis. All analyses were performed by double-blinded quantification.

### Quantifications of immunolabeled LDLR and CD36

Sciatic nerve sections (14 μm) were stained with an antibody against *LDLR* and *CD36* (Supplemental Table 1). On average, 10 images were measured for every mouse. Intensity expression was calculated using ImageJ software and normalized to the DAPI intensity of each image. All analyses were performed by double-blinded quantification.

### Electron microscopy

Mice were perfused with 20 mL 1× PBS followed by 15 mL of 4% PFA (Electron Microscopy Sciences, 15172) diluted in PHEM buffer (0.1M; pH 7.4) (Electron Microscopy Sciences, 11163). Sciatic and sural nerves were dissected and fixed in 4% PFA + 2.5% glutaraldehyde (Acros Organics, Thermo Fisher Scientific) diluted in PHEM buffer overnight at 4°C. Tissues were stored in 0.5% glutaraldehyde/PHEM buffer at 4°C until use. After being washed in 0.2M PBS buffer, the nerves were incubated with 0.5% osmic acid + 0.8% potassium hexacyanoferrate trihydrate in 0.1M phosphate buffer for 120 minutes at RT. After 2 rinses in PHEM buffer, the nerves were dehydrated in a graded series of ethanol solutions (30%–100%). The nerves were embedded in EmBed 812 using an Automated Microwave Tissue Processor for Electronic Microscopy, Leica EM AMW. Thin sections (70 nm; Leica-Reichert Ultracut E) were collected at different levels of each block. These sections were counterstained with 1.5% uranyl acetate in 70% ethanol and lead citrate before observation using a Tecnai F20 transmission electron microscope at 120 kV in the Institut des Neurosciences de Montpellier (Université de Montpellier, Montpellier, France).

The axonal diameter was manually and blindly measured using the MyelTracer software (87). Mitochondria were manually and double-blindly quantified. Transmission electron microscopy (TEM) images of sciatic nerve sections were acquired at (×8,500, ×10,000) magnifications of 20 randomly selected fields.

### Toluidine blue staining and myelinated fiber analysis

Slides were stained with 1% toluidine blue solution containing 2% borax, washed with water, and then mounted in Entellan mounting medium (EMS). Reconstituted tiles of the whole nerves were imaged at ×63 magnification. To determine *g*-ratio, images were cropped into random fields. Axon and fiber (axon + myelin) diameters for at least 1,162 axons per genotype (on average, about 300 axons per mouse) were calculated using MyelTracer software (87). All quantifications were performed by an individual blinded to the animal genotype.

### Lipid droplet staining

For lipid droplets staining, sections were fixed in 4% PFA, washed with 1× PBS, and incubated for 45 minutes at 20°C with 1 μg/mL of the fluorescent neutral lipid stain Bodipy 493/503 (Invitrogen). After washing, sections were counterstained with DAPI and mounted in vectashield (Vector Laboratories).

### Proteomics

Nerves from WT mice (*n* = 4) and *PCSK9-*KO mice (*n* = 3) were pooled until reaching 10 mg and lysed with RIPA buffer (50 mM Tris-HCl [pH 7.2], 5 mM EDTA, 0.1%SDS) and Halt Protease Inhibitor Cocktail (Thermo Fisher Scientific) for 60 minutes at 4°C with constant shaking. Next, samples were centrifuged at 13,000*g* for 5 minutes, the supernatant containing the protein extract was collected, and the total protein concentration was determined (BCA Protein Assay Kit, Thermo Fisher Scientific). Samples were reduced with DTT (15 mM), and protein alkylation was then performed at room temperature (RT), by incubating with iodoacetamide (final concentration 15 mM), for 30 minutes in the dark. Trypsin digestion (enzyme/protein ratio of 1:20), was performed overnight at 37°C, and the tryptic peptide digests were resuspended in 7 μL of 4% acetonitrile (ACN) in 0.1% TFA and analyzed by nano-LC using a Thermo Fisher Scientific Ultimate 3000 series NCS-3500 RS coupled with NSI-Q-Orbitrap mass spectrometer (Q Exactive Plus, Thermo Fisher Scientific). In total, 5 μL of sample (each sample was injected in triplicate) as separated on a LC-EASY-spray C18 column (2.6 μm, 100 Å, 75 μm × 25 cm, Thermo Fisher Scientific). The mass spectrometry (MS) analysis was performed with the following conditions: spray voltage, 2 kV; heated capillary temperature, 275°C; and S-lens RF level, 30%. Mass spectra were acquired with XCalibur 4.2.47 software (Thermo Fisher Scientific) and registered in data-dependent acquisition with the mass spectrometer operating in positive mode. Survey full scan mass spectra were acquired in the 350–2,000 m*/z* range at a resolving power of 70,000 (at *m/z* 400) with automatic gain control (AGC) target of 1 × 10^6^ and maximum injection time (max IT) of 120 ms. Injection of blank (4% ACN in 0.1% TFA) was performed before and after samples to prevent carryover. The Orbitrap performance was evaluated weekly, and external calibration of the mass spectrometer was performed prior to analysis with an LTQ ESI positive ion calibration solution (Pierce).

### Protein identification and quantification

Raw MS data were automatically processed using Proteome discoverer software (version 2.2.2.2.0, Thermo Fisher Scientific) for protein identification and quantification. MS and MS/MS spectra were searched against the UniProt mouse reference proteome database with canonical and isoform sequences. The database search was performed with the following parameters: oxidized methionine and protein N-terminal acetylation were set as variable modifications, and Cysteine carbamidomethylation was set as a fixed modification. Trypsin was set as enzyme-specific, and 2 missed cleavages were allowed. A mass tolerance of 10 ppm was used for precursor ions and 0.02 Da for-product ions. The FDR was fixed at 1% at the level of proteins and peptides using a target-reversed decoy database search strategy. A minimum of 1 unique peptide sequence with a Sequest score (XCorr) ≥ 2 was used. For peptides with an XCorr < 2, identification was confirmed by manual interpretation of the corresponding MS/MS spectrum.

### Nerve sample lipid extraction and lipidomic analysis

Nerve sample was weighted, snap-frozen crushed with a Potter tissue grinder, and then homogenized in ice-cooled PBS. (0.1×) with a proportion of 1:300 (mass:volume [m:v]). Protein assay on each nerve sample homogenate was performed using bicinchoninic acid method with bovine serum albumin as a standard. Nerve lipids were extracted by mixing 10 μL of homogenate with 100 μL of 10 mM ammonium formate in butanol:methanol (1:1, v:v). AC internal standard [palmitoyl-L-carnitin-(N-methyl-d3)] was added to each sample at a concentration of 200 pmol. Samples were vortexed thoroughly, followed by sonication in a sonicator bath for 1 hour at 20°C. Subsequently, centrifugation was performed at 16,200*g* for 10 minutes at 20°C. Finally, 90 μL of lipid extract was transferred to vials with glass inserts and directly analyzed by MS.

To measure individual nerve lipid species, a shotgun lipidomic approach using ultra-high–performance liquid chromatograph coupled to a HESI-orbitrap mass spectrometer (Q-Exactive Plus, Thermo Fisher Scientific) was employed. Briefly, 1 mL of sample was separated on a ZORBAX eclipse plus C18 column (2.1 mm × 100 mm, 1.8 μm; Agilent) with the thermostat set at 45°C. Lipids were eluted with a gradient mixture of water/ACN/isopropanol (5:3:2, v:v:v) containing 10 mM ammonium formate (A) and water/ACN/isopropanol (1:9:90, v:v:v) containing 10 mM ammonium formate (B) at a flow rate of 0.4 mL/min, with 15% B at 0 to 2.5 minutes, 50% B at 2.5–2.6 minutes, 57% B at 2.6–9 minutes, 70% B at 9–9.1 minutes, 93% B at 9.1–11 minutes, 96% B at 11–11.1 minutes, and 15% B at 11.1–16 minutes.

The following mass spectrometer optimized parameters were applied: spray voltage 3.5 kV, capillary temperature 350°C, sheath gas flow rate 45 L/min, gas aux gas flow rate 10, and S-lens RF level: 40%. Mass spectra were registered in positive ion mode with XCalibur 4.2 software (Thermo Fisher Scientific). Data were processed with the Skyline 24.1 software (MacCoss Lab) by using a house-made database.

Lipid abundances were calculated by plotting the peak intensity of the lipid species against the corresponding lipid standard concentrations (0.02–10 mM). The total AC class concentration was calculated on the basis of the sum of the individual AC species that were detected.

### Statistics

Graph Pad-Prism 8 (GraphPad Software Inc.) was used for graphic presentation and statistical analysis. Results are expressed as the mean per group ± SD. We considered *P* < 0.05 as significant. For a 2-group comparison, a 2-tailed *t* test was used. For multiple comparisons, a 1-way ANOVA followed by Tukey’s multiple-comparison test was used. For the integration of the proteome for functional analysis, protein abundance was calculated with the generation of spectral feature by the node Feature Finder Multiplex followed by Protein Inference Algorithms–assisted (PIA-assisted) FDR-multiple scores estimation and filtering (combined FDR score < 0.01), their ID mapping and combination with peptide IDs, their subsequent alignment, grouping, and normalization. The Database for Annotation, Visualization, and Integrated Discovery (DAVID) platform was used for gene ontology (GO) enrichment analysis. GO was performed in our protein Label-Free Quantification (LFQ) dataset, and results for significant terms associated with the gene set were selected based on the FDR < 0.05.

### Study approval

All in vivo experiments were conducted in accordance with the European Community Guidelines for the Use of Animals in Research (86/609/EEC and 2010/63/EU) and approved by the local Ethics Committee for animal experimentation (APAFIS#3209- 2015111215451823v2).

### Data availability

The corresponding author will provide all requested materials, data sets, and protocols, without restriction, upon request. Values for all data points in graphs are reported in the Supporting Data Values file.

## Author contributions

AKJ and SB designed the studies. AKJ, APR, GR, BV, CP, MB, KC, and PR carried out the experiments. AKJ, GCL, and SB interpreted the data. AKJ, OM, GCL, and SB drafted the manuscript, and participated in its writing and editing.

## Supplementary Material

Supplemental data

Unedited blot and gel images

Supporting data values

## Version 1 05/08/2025

In-Press Preview

## Version 2 06/23/2025

Electronic publication
