# Supplementary material for: PCSK9 deficiency promotes the development of peripheral neuropathy
Source: JCI Insight. 2025 May 8;10(12):e183786. doi: 10.1172/jci.insight.183786 (PMC12220952; doi:10.1172/jci.insight.183786)
Supplement: Supplemental data [file jciinsight-10-183786-s217.pdf]

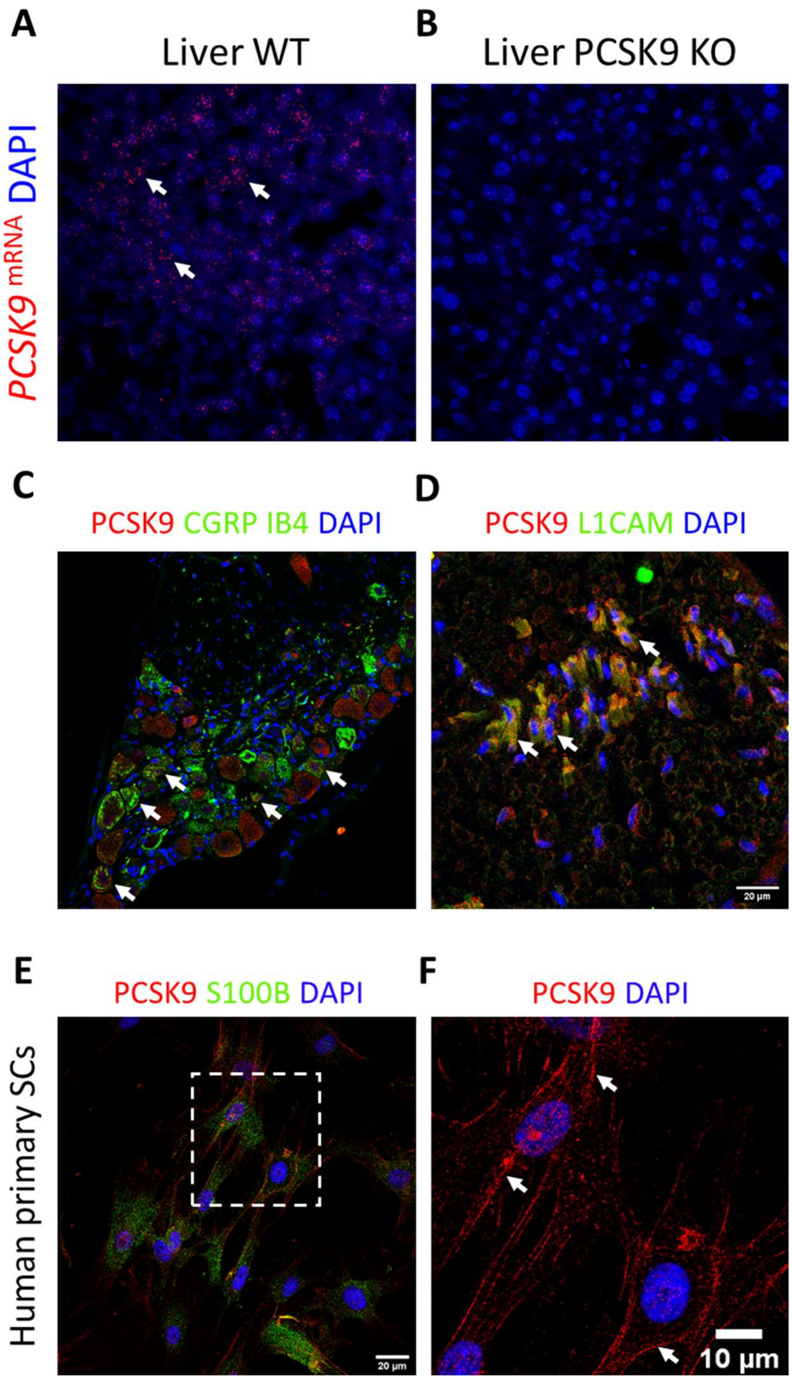

Supplemental Figure 1. ***PCSK9*** **expression in the liver, DRG and primary SCs.**

(A) *PCSK9* mRNA expression in mouse liver of control (*WT*). **(B)** *PCSK9* mRNA expression in mouse liver of *PCSK9 KO* mice. **(C)** *PCSK9* expression in non- myelinated sensory neurons expressing *CGRP* and *IB4*. **(D)** *PCSK9* expression in Schwann cell subtypes in mouse sciatic nerve, showing colocalization of *PCSK9* with *L1CAM* positive non–myelinating Schwann cells. **(E)** Expression of *PCSK9* in Human primary Schwann cells culture. (**F**) High magnification of the dashed square in (E). Arrows indicate *PCSK9* expression.

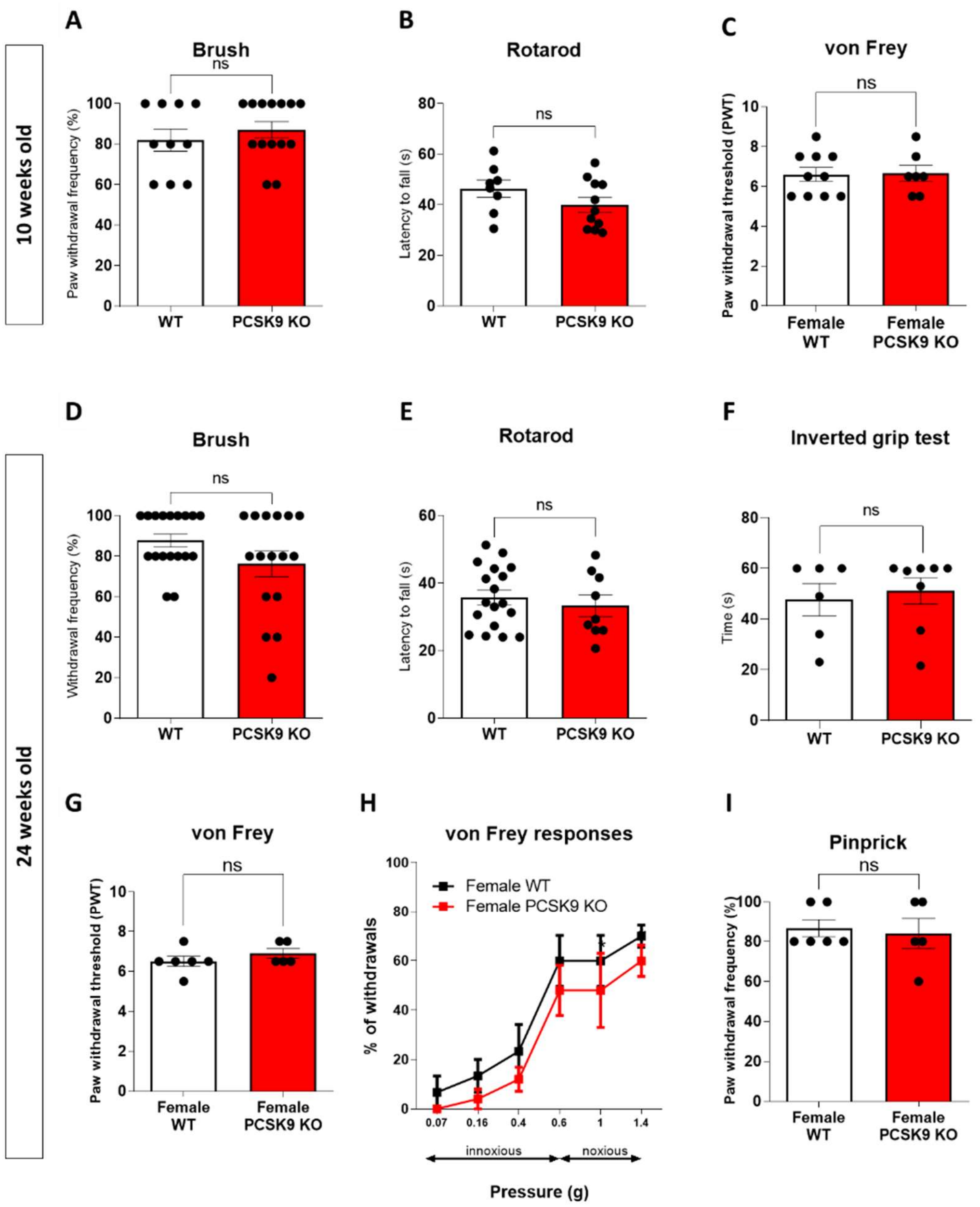

Supplemental Figure 2. Behavioral analysis of *PCSK9 KO* mice

(A) Dynamic touch sensation evaluated with the brush test in 10-week-old *WT* (n=10) and *PCSK9 KO* (n=14) mice. **(B)** Gross motor coordination evaluated with the rotarod test in 10-week-old *WT* (n=8) and *PCSK9 KO* (n=11) mice. **(C)** Mechanical pain sensation evaluated with the von Frey test in 10-week-old female *WT* (n=10) and *PCSK9 KO* (n=7) mice. **(D)** Dynamic touch sensation evaluated with the brush test in 24-week-old *WT* (n=18) and *PCSK9 KO* (n=16) mice. **(E)** Gross motor coordination evaluated with the rotarod test in 24-week-old *WT* (n=18) and *PCSK9 KO* (n=9) mice. **(F)** Assessment of grip strength with the inverted grip test in 24-week-old *WT* (n=6) and *PCSK9 KO* mice (n=8). **(G)** Mechanical pain sensation evaluated with the von Frey test in 24-week-old female *WT* (n=6) and *PCSK9 KO* (n=5) mice. **(H)** Mechanical pain sensation evaluated with the von Frey test presented as withdrawal percentage in 24-week-old female *WT* (n=6) and *PCSK9 KO* (n=5) mice. **(I)** Acute mechanical pain sensation evaluated with the pinprick test in 24-week-old female *WT* (n=6) and *PCSK9 KO* (n=5) mice. Data are represented as mean ± SEM and statistically analyzed by unpaired t test. $P > 0.05$ (ns).

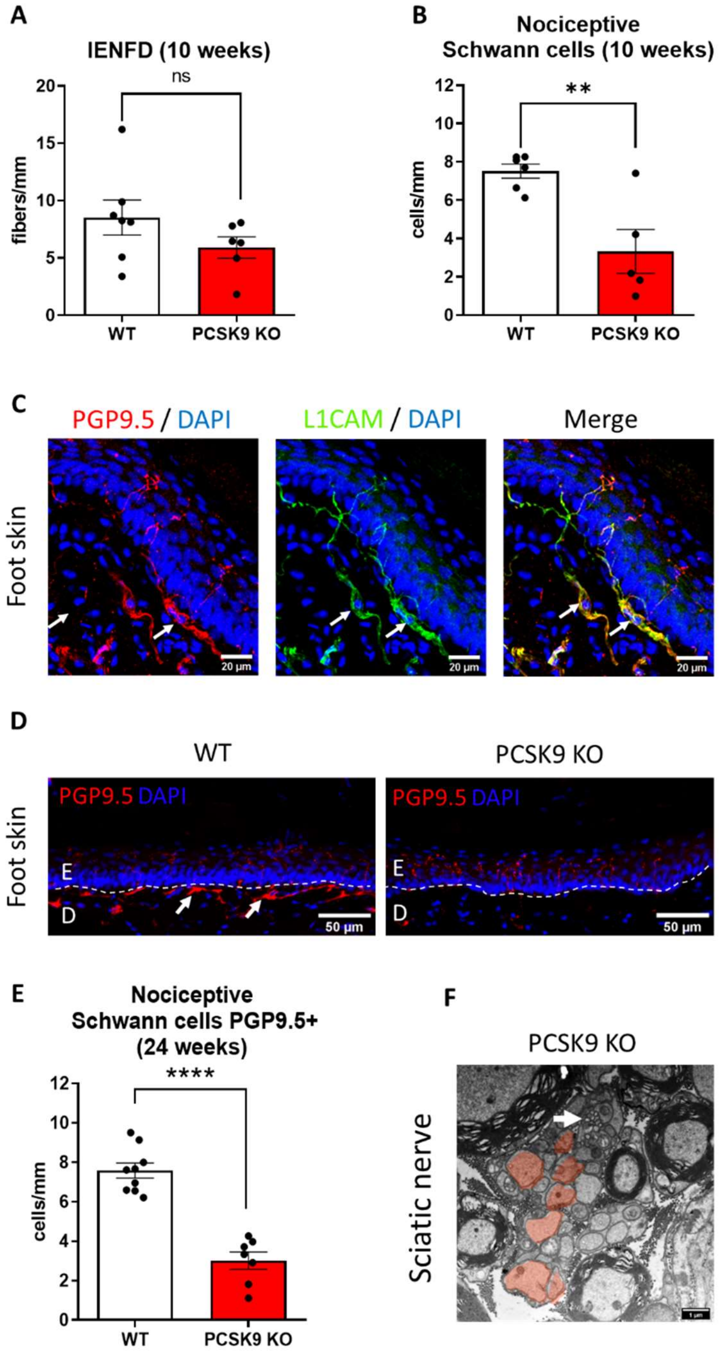

Supplemental Figure 3. IENFD and nociceptive SC in *PCSK9 KO* mice

(A) Quantification of IENFD in 10 weeks old *WT* (n=7) and *PCSK9 KO* (n=6) mice. **(B)** Quantification of numbers of nociceptive Schwann cells in 10 weeks old *WT* (n=6) and *PCSK9 KO* (n=5) mice. **(C)** Images of terminal nociceptive Schwann cells with colocalization between *PGP9.5* and *L1CAM*. **(D)** Representative images of nociceptive Schwann cells identified by immunostaining against *PGP9.5*/DAPI in the foot skin of *WT* and *PCSK9 KO* mice. **(E)** Quantification of the number of nociceptive Schwann cells in *WT* (n=9) and *PCSK9 KO* (n=7) mice. **(F)** Electron microscopy picture of sciatic nerve showing structural abnormalities in axonal circularity in *PCSK9 KO* mice (red highlight). Arrow shows vacuoles inside the nerve. Data are represented as mean ± SEM and statistically analyzed by unpaired t test. $P > 0.05$ (ns), $^{**}P < 0.01$, $^{****}P \leq 0.0001$.

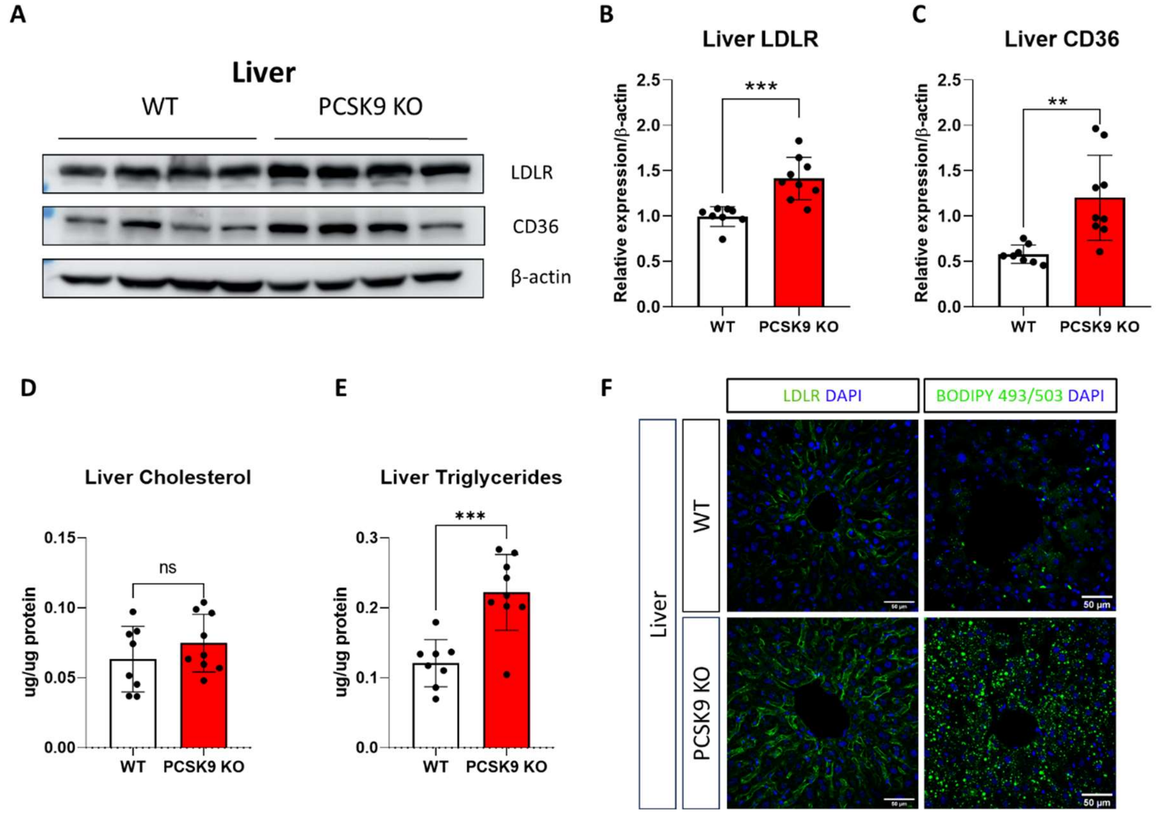

Supplemental Figure 4. ***LDLR*** **and** ***CD36*** **expression in the liver of control and** ***PCSK9 KO*** **mice.**

(A) *LDLR* and *CD36* expression in the liver by immunoblotting. **(B)** Quantification of *LDLR* liver expression in 24-week-old *WT* (n=9) and *PCSK9 KO* (n=9). **(C)** Quantification of *CD36* liver expression in 24-week-old *WT* (n=8) and *PCSK9 KO* (n=9). **(D)** Cholesterol levels in the liver of 24-week-old *WT* (n=8) and *PCSK9 KO* (n=9) mice. **(E)** Triglycerides levels in the liver of 24-week-old *WT* (n=8) et *PCSK9 KO* (n=9) mice. **(F)** *LDLR* expression and bodipy staining in section of liver from 24-week-old *WT* and *PCSK9 KO* mice. Data are represented as mean ± SEM and statistically analyzed by unpaired t test. $P > 0.05$ (ns), $^{**}P < 0.01$, and $^{***}P < 0.001$.

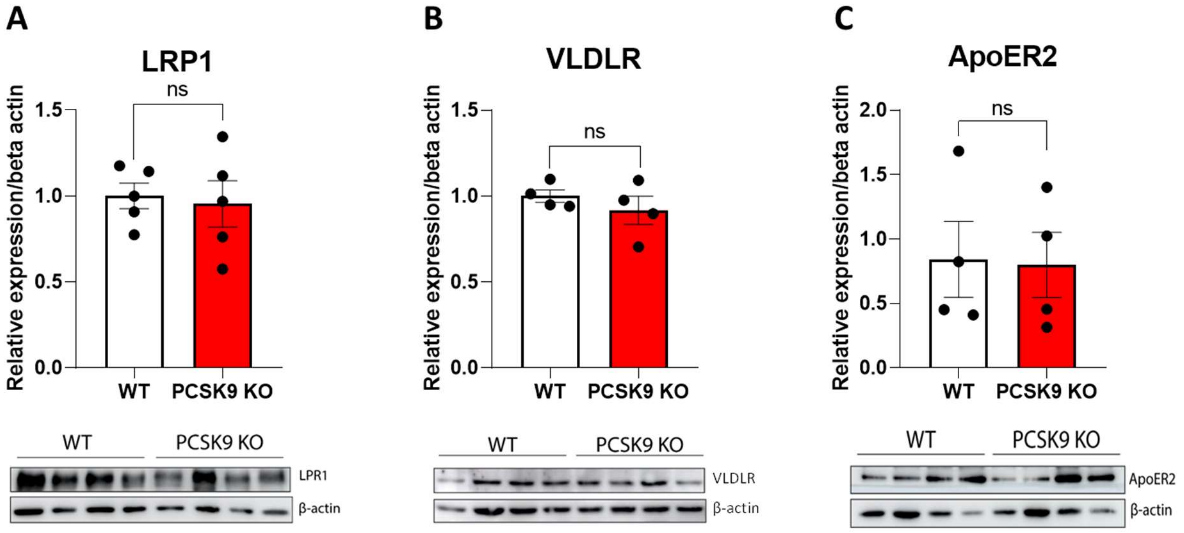

Supplemental Figure 5. *LRP1*, *VLDLR* and *ApoER2* expression in the sciatic nerve of control and *PCSK9 KO* mice

(A) Quantification of *LRP1* expression in the sciatic nerve of 24-week-old *WT* (n=5) and *PCSK9 KO* (n=5) mice. **(B)** Quantification of *VLDLR* expression in the sciatic nerve of 24-week-old *WT* (n=5) and *PCSK9 KO* (n=5). **(C)** Quantification of *ApoER2* expression in the sciatic nerve of 24-week-old *WT* (n=5) and *PCSK9 KO* (n=5). Data are represented as mean ± SEM and statistically analyzed by unpaired t test. $P > 0.05$ (ns).

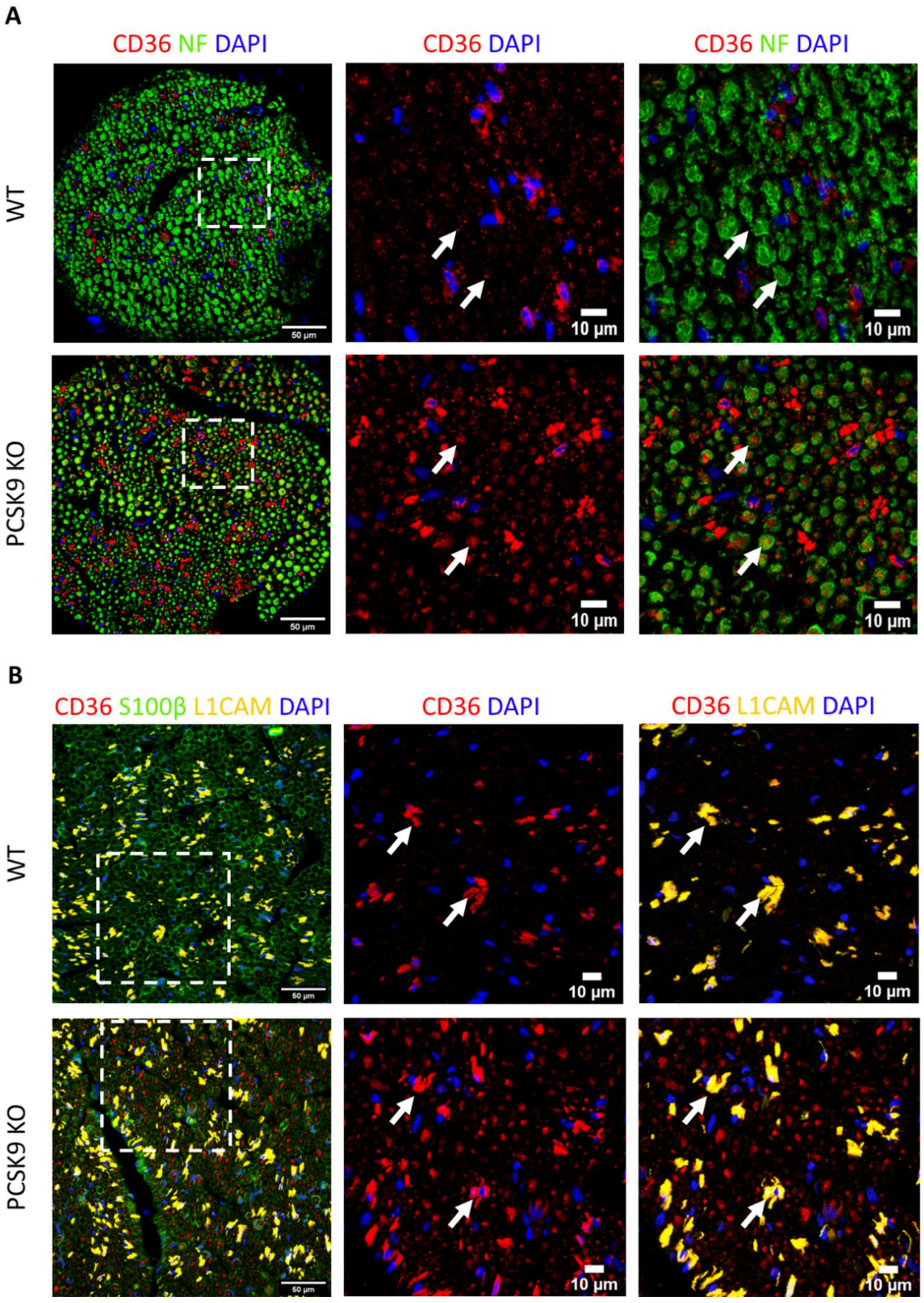

Supplemental Figure 6. *CD36* expression in the sciatic nerve of control and *PCSK9 KO* mice.

(A) Transverse section of mouse sciatic nerve of 24-week-old *WT* and *PCSK9 KO* labeled with an antibody against *CD36* (red) and neurofilament (*NF*) (green) as well as nuclear DAPI staining (blue). **(B)** Triple immunostaining of transverse section mouse sciatic nerve of 24-week-old *WT* and *PCSK9 KO* with antibody against *CD36* (red), *S100B* a pan-Schwann cell marker (green) and *L1CAM* a non-myelinating Schwann cell marker (yellow) as well as nuclear DAPI staining (nuclear staining blue). (Arrows show the colocalization of *CD36* with the different markers).

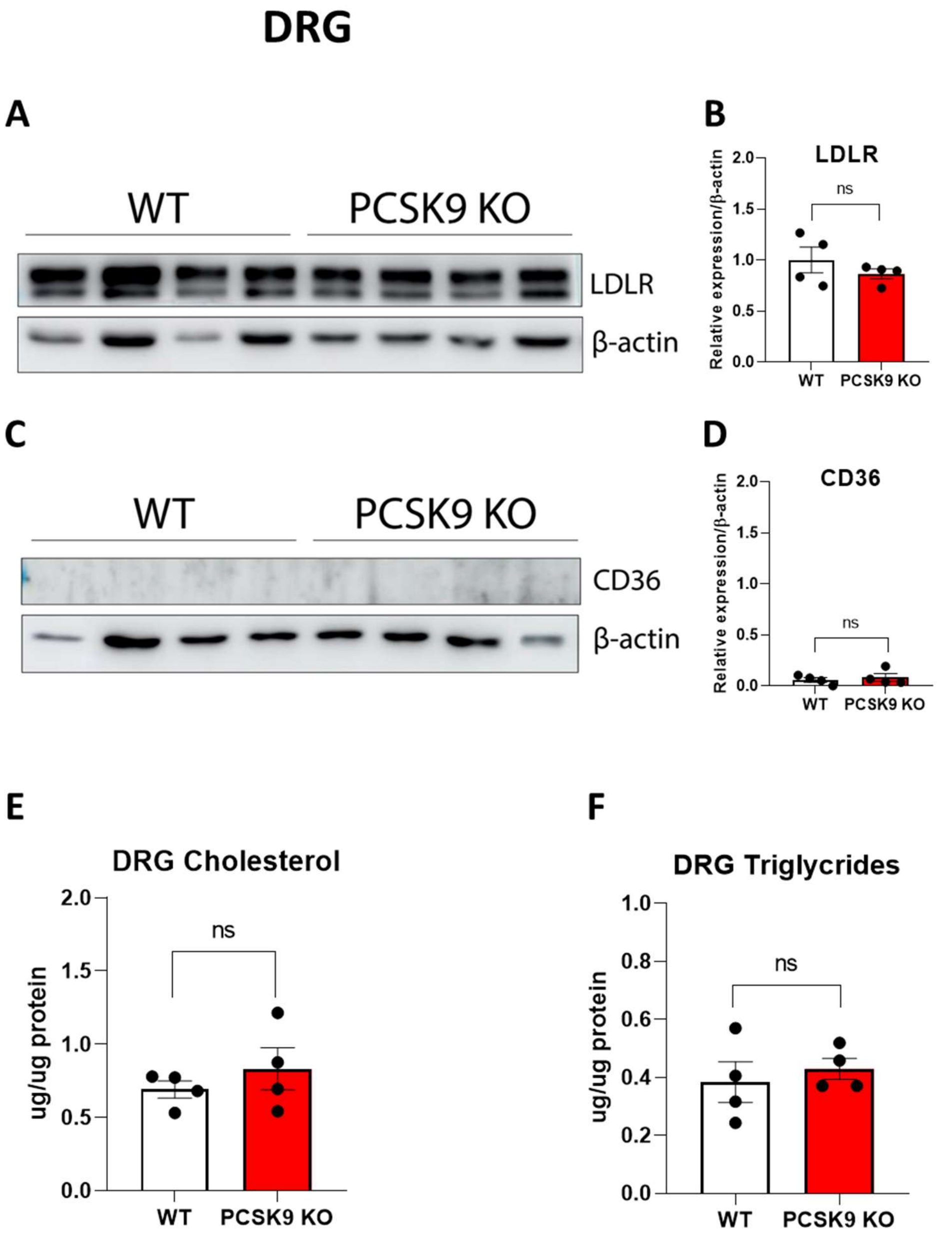

Supplemental Figure 7. *LDLR* and *CD36* expression in DRG of control and *PCSK9 KO* mice.

(A) Immunoblot of *LDLR* expression in DRG of 24-week-old *WT* and *PCSK9 KO* mice. **(B)** Quantification of *LDLR* expression level in DRG of *WT* (n=4) and *PCSK9 KO* (n=4) mice. **(C)** Immunoblot of *CD36* expression in DRG of 24-week-old *WT* and *PCSK9 KO* mice. **(D)** Quantification of *CD36* expression level in DRG of *WT* (n=4) and *PCSK9 KO* (n=4) mice. **(E)** Quantification of cholesterol level in DRG of 24 weeks *WT* (n=4) and *PCSK9 KO* (n=4) mice. **(F)** Quantification of triglycerides levels in DRG of 24-weeks-old *WT* (n=4) and *PCSK9 KO* (n=4) mice. Data are represented as mean ± SEM and statistically analyzed by unpaired t test. $P > 0.05$ (ns).

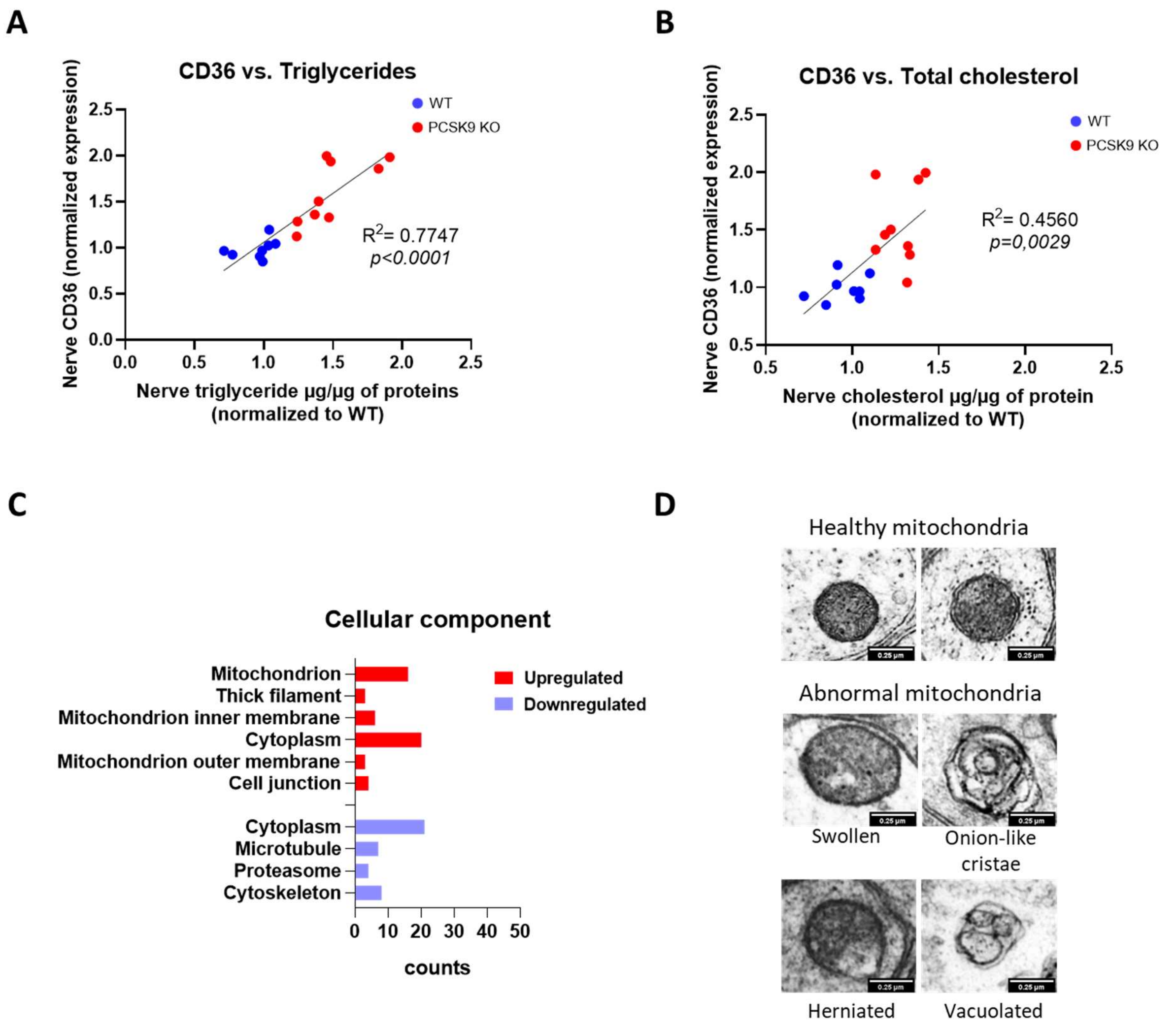

Supplemental Figure 8. Correlation between *CD36* expression and nerve triglyceride and cholesterol concentration.

(A) Scatter plot displaying the correlation between nerve *CD36* expression and nerve triglyceride levels in *WT* (n=8) and *PCSK9 KO* (n=9). **(B)** Scatter plot displaying the correlation between nerve *CD36* expression and nerve cholesterol levels in *WT* (n=8) and *PCSK9 KO* (n=9). **(C)** Gene ontology annotation of the top upregulated (red) and downregulated (blue) in terms of molecular function and (D) cellular component sorted by P-value. **(D)** Representative images of healthy mitochondria and abnormal mitochondria.

Target antigen	Vendor or source	Catalog #	Experiments
**Rabbit anti human pro-PCSK9 N-terminal domain**	**Abcam**	**ab135647**	**IF**
**Goat anti human PCSK9 C-terminal domain**	**Abcam**	**ab28770**	**IF**
**Rabbit anti-mouse PCSK9**	**Cayman**	**10240**	**IF**
**Mouse anti-S100β**	**Sigma-Aldrich**	**S2532**	**IF**
**Rat anti- L1CAM**	**Merck milipore**	**MAB5272**	**IF**
**Goat anti-LDLR**	**R&D**	**AF2255**	**IF,WB**
**Mouse anti-ApoER2**	**Abnova**	**H00007804-M01**	**IF,WB**
**Rabbit anti-LRP1**	**Abcam**	**ab92544**	**IF,WB**
**Goat anti-VLDLR**	**R&D**	**AF2258**	**IF,WB**
**Chicken anti-NF**	**Merck milipore**	**AB5539**	**IF**
**Rabbit anti-β-actin**	**Abcam**	**ab8227**	**WB**
**Rabbit anti-PGP9.5**	**Merck milipore**	**AB1761**	**IF**
**Rabbit anti-CD36**	**Abcam**	**ab124515**	**IF,WB**
**Donkey anti-Rabbit Alexa 594**	**ThermoFisher**	**A-21207**	**IF**
**Donkey anti-Mouse Alexa 594**	**ThermoFisher**	**A-21203**	**IF**
**Donkey anti-Rat Alexa 488**	**ThermoFisher**	**A-21208**	**IF**
**Donkey anti-Goat Alexa 594**	**ThermoFisher**	**A-11058**	**IF**
**Donkey anti-Chicken Alexa 488**	**Invitrogen**	**A78948**	**IF**
**Donkey Anti-Goat IgG H&L (HRP)**	**Abcam**	**ab205723**	**WB**
**Peroxidase AffiniPure™ Donkey Anti-Rabbit IgG (H+L)**	**Jackson ImmunoResearch**	**711-035-152**	**WB**
**Peroxidase AffiniPure™ Donkey Anti-Mouse IgG (H+L)**	**Jackson ImmunoResearch**	**715-035-150**	**WB**

Table 1: List of all antibodies used (IF: immunofluorescence, WB: western blot)
