# Supplementary material for: PCSK9 deficiency promotes the development of peripheral neuropathy
Source: JCI Insight. 2025 May 8;10(12):e183786. doi: 10.1172/jci.insight.183786 (PMC12220952; doi:10.1172/jci.insight.183786)

Full unedited blot for Figure 5A

LDLR

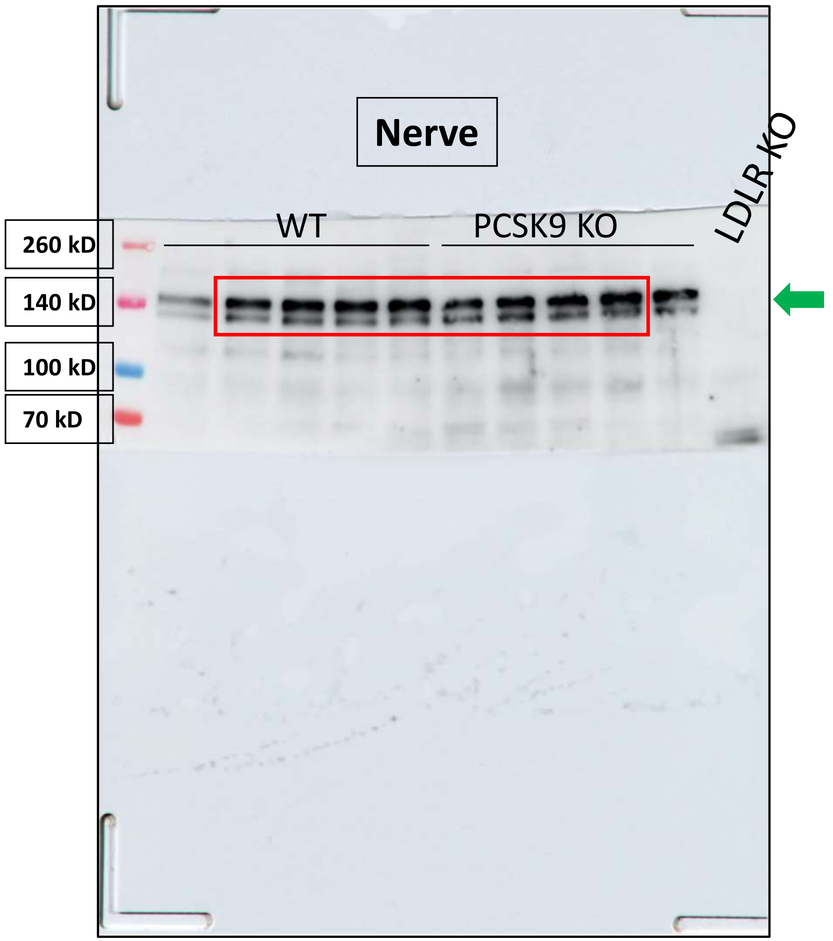

β-actin

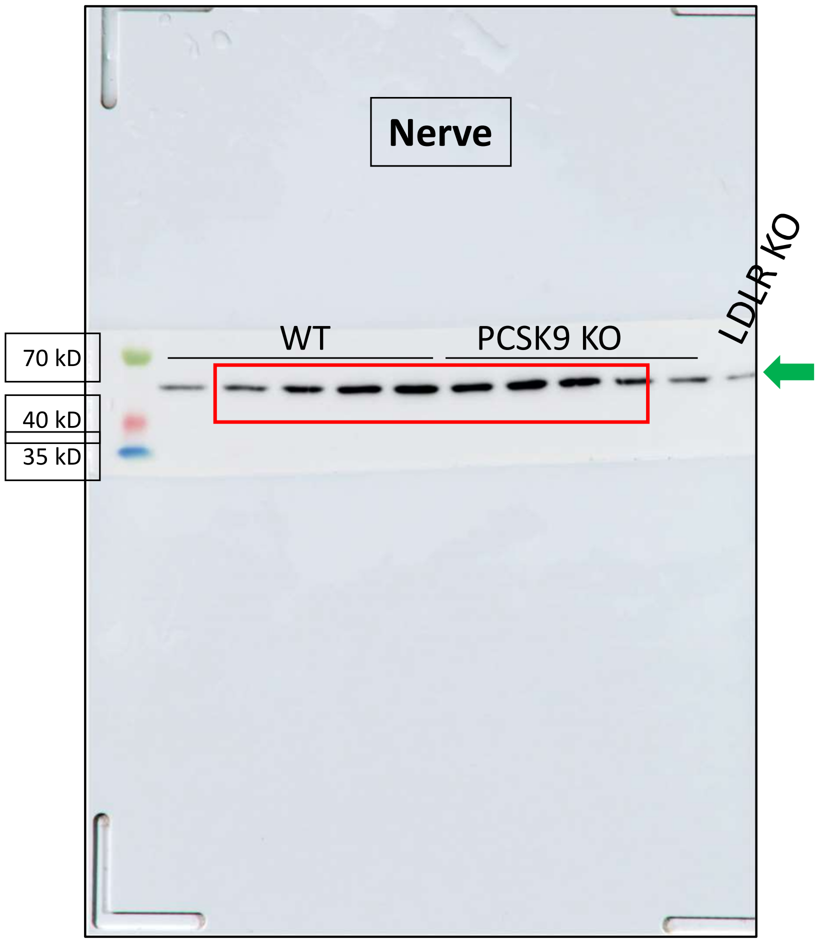

Full unedited blot for Figure 5E

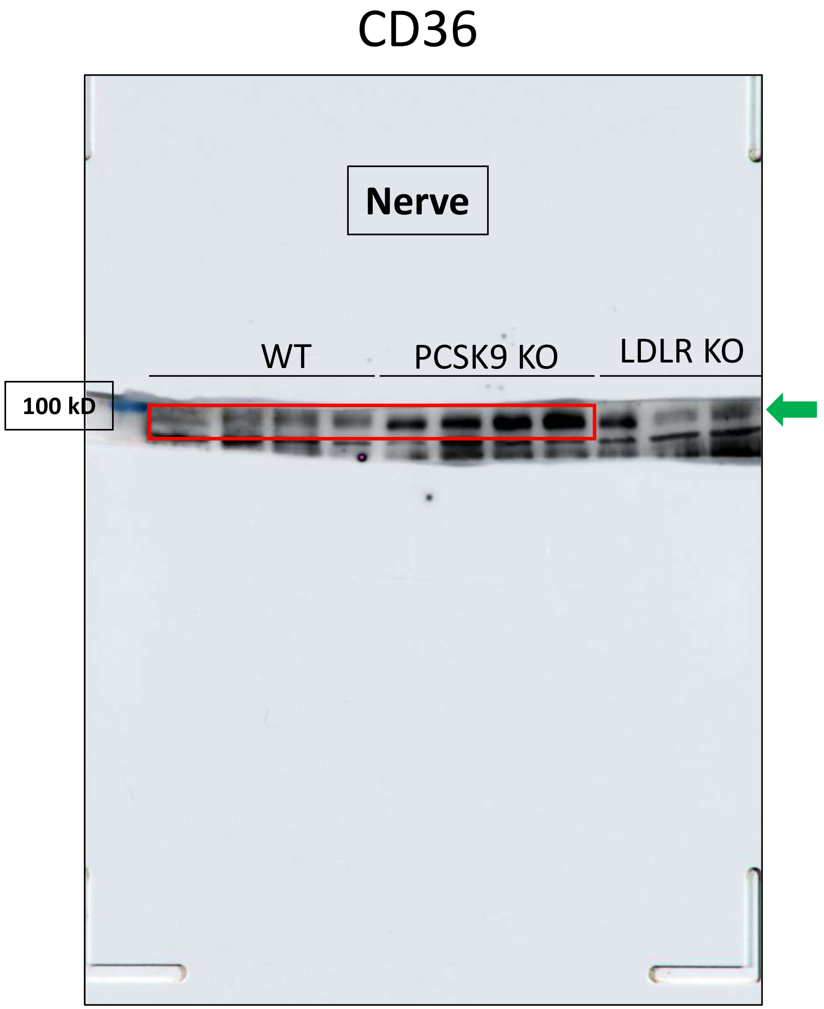

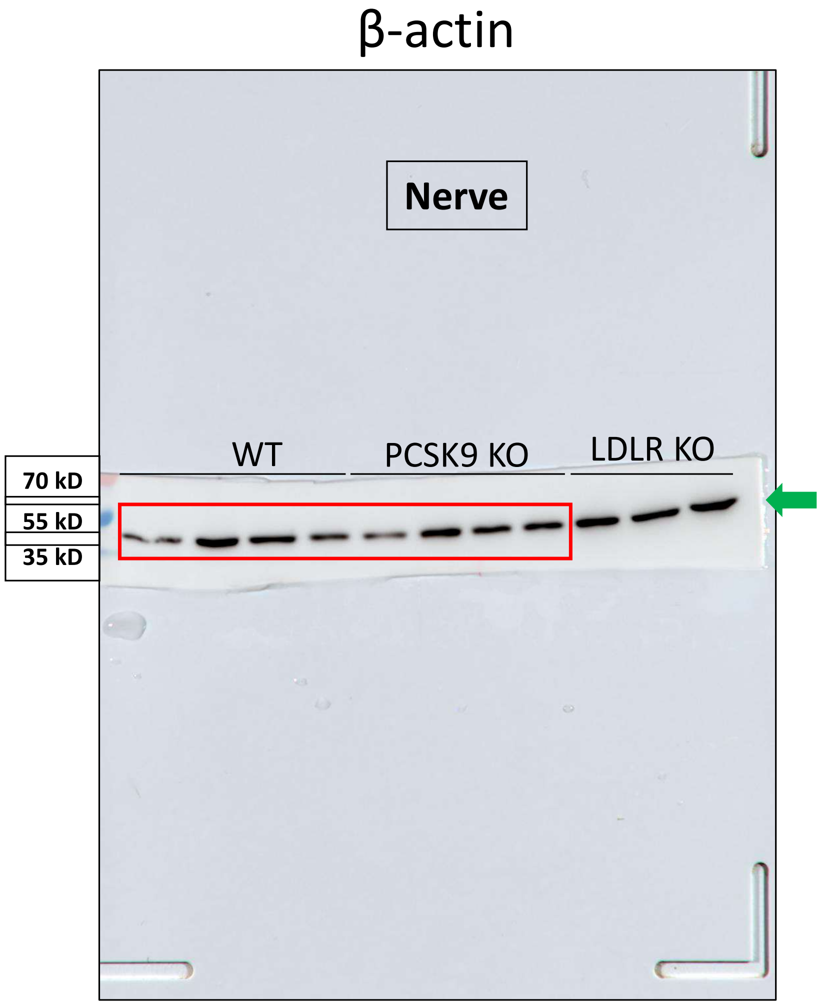

Full unedited blot for Supplementary Figure 4A

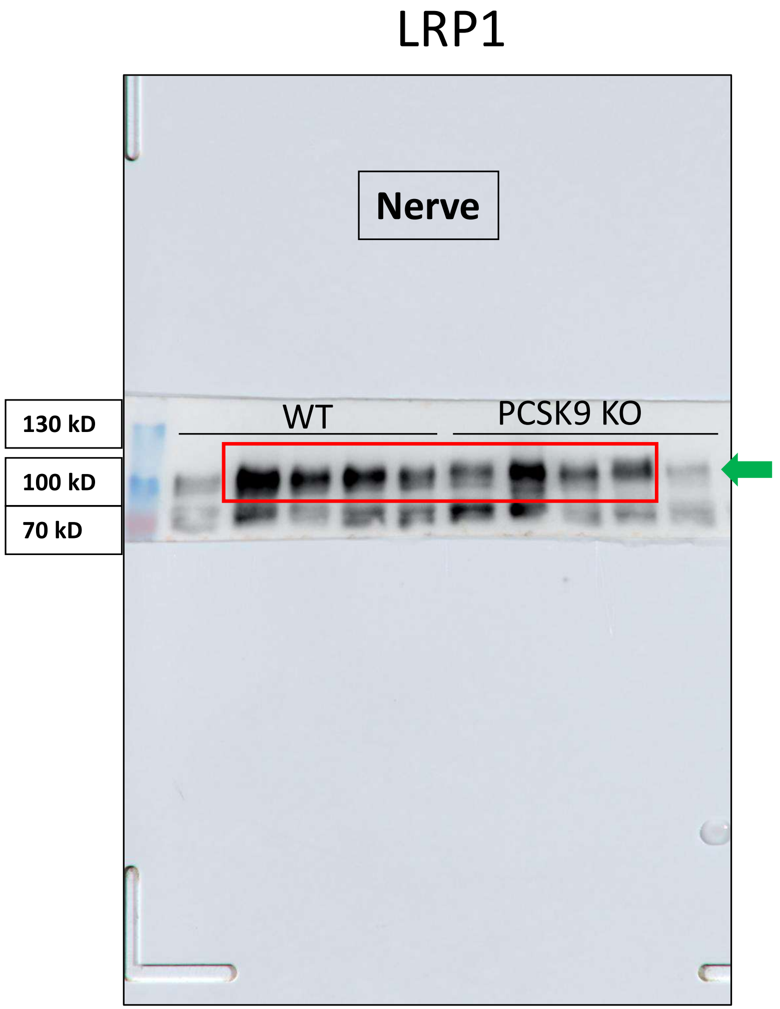

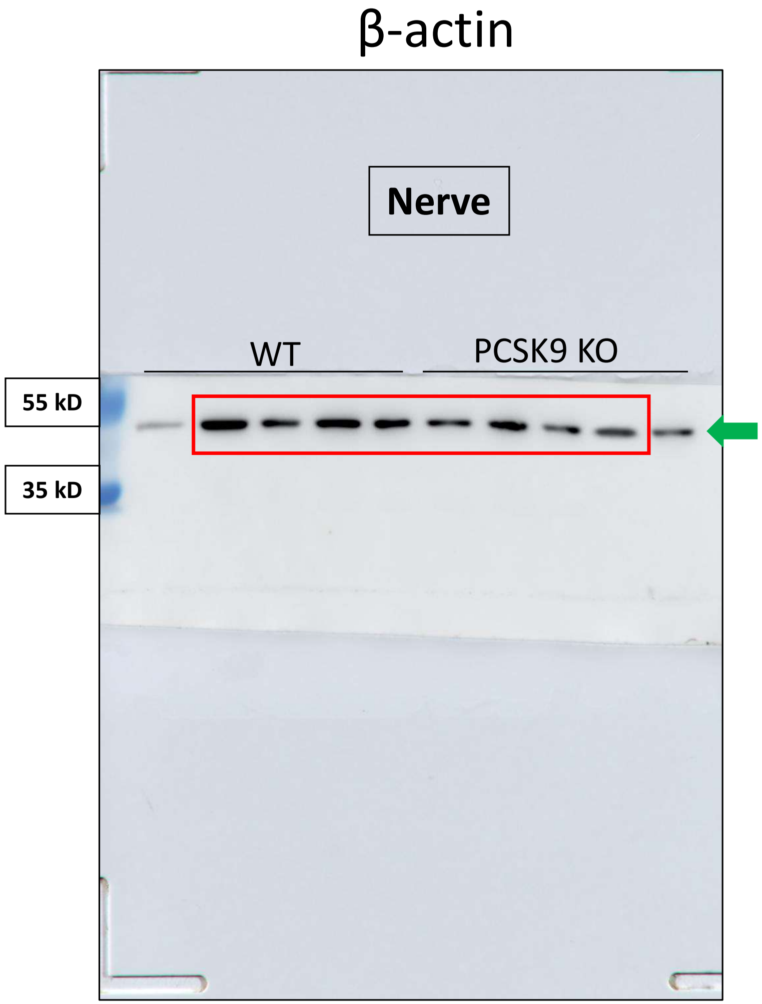

Full unedited blot for Supplementary Figure 4B

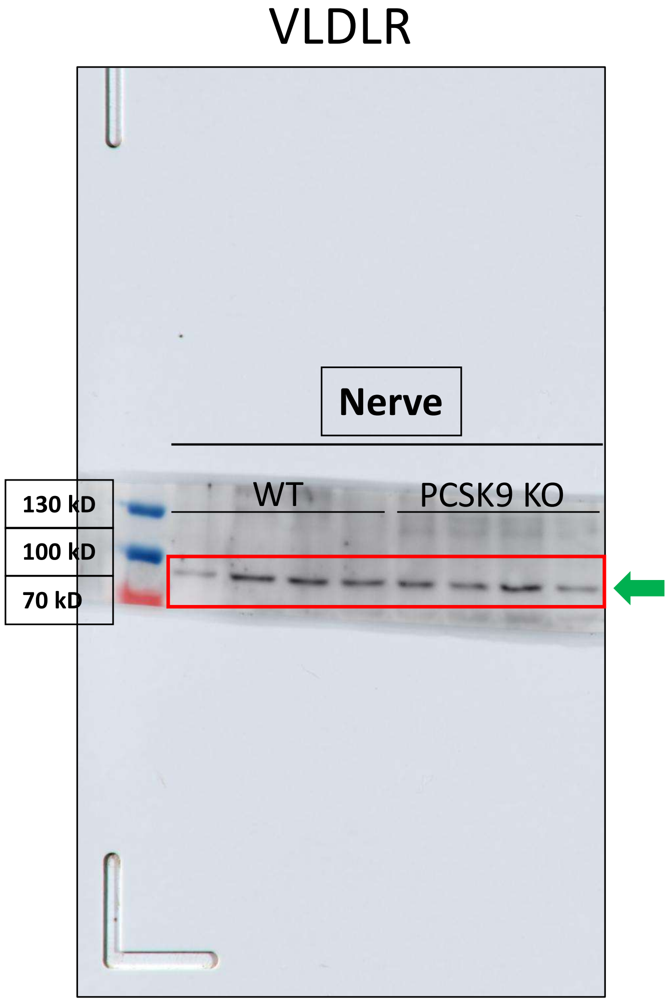

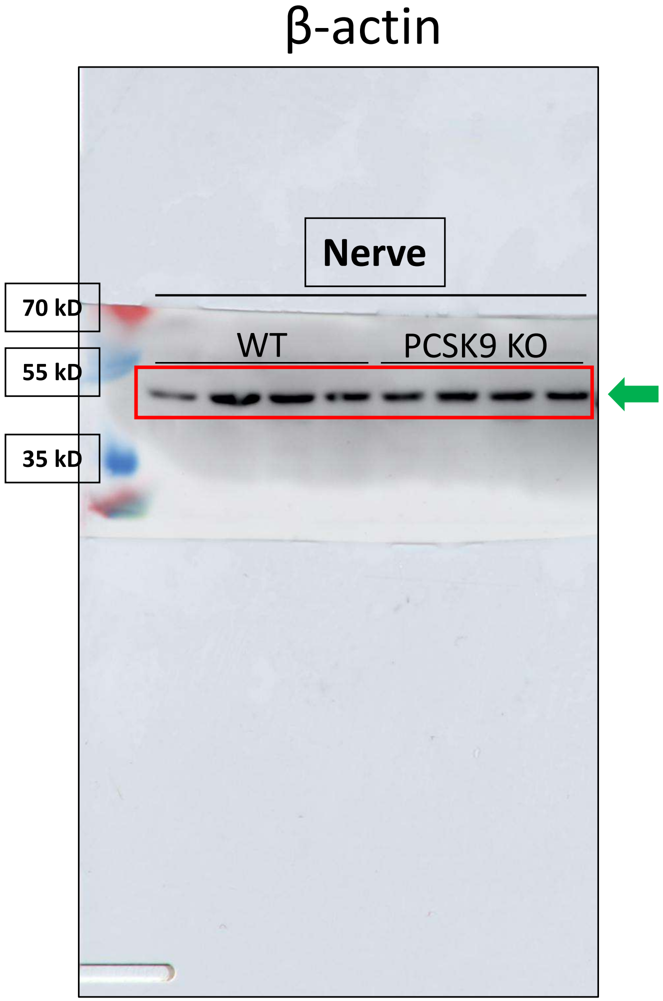

Full unedited blot for Supplementary Figure 4C

ApoER2

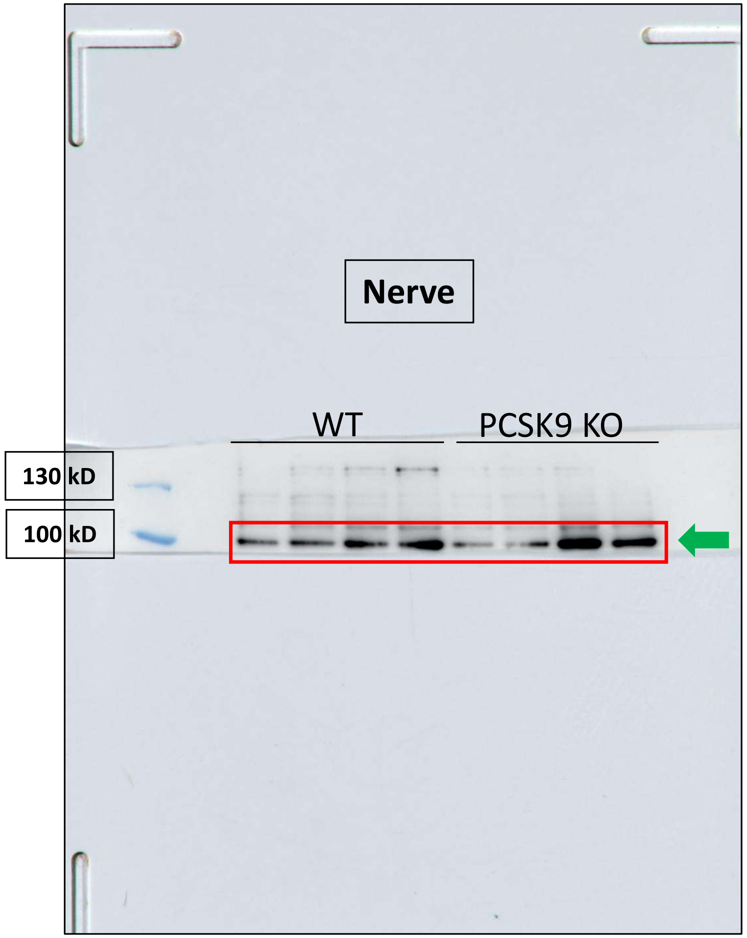

β-actin

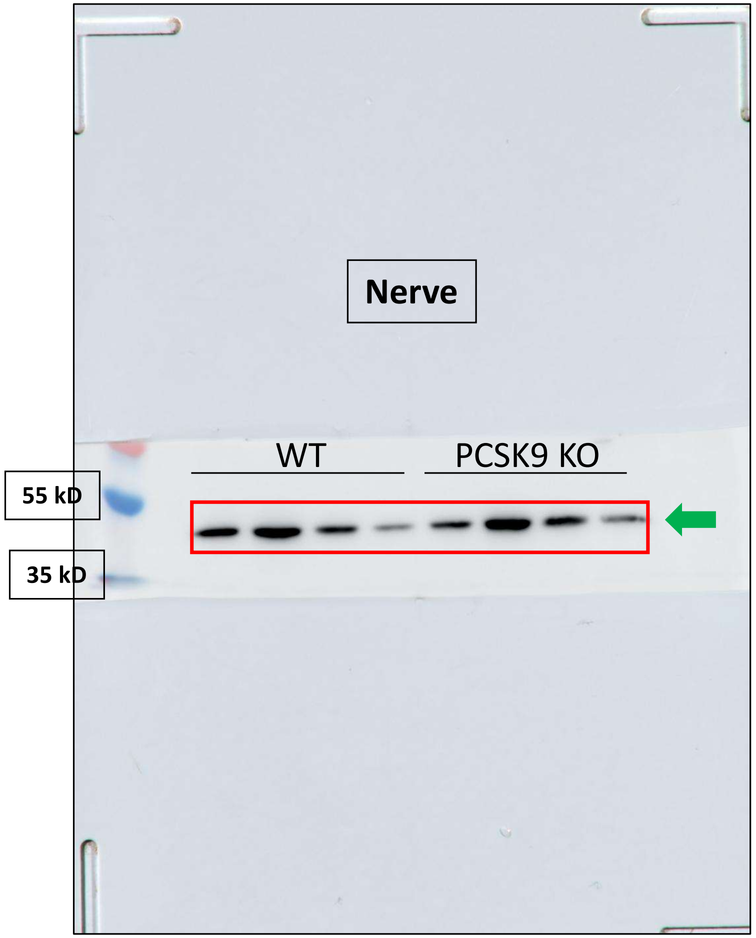

Full unedited blot for Supplementary Figure 5A

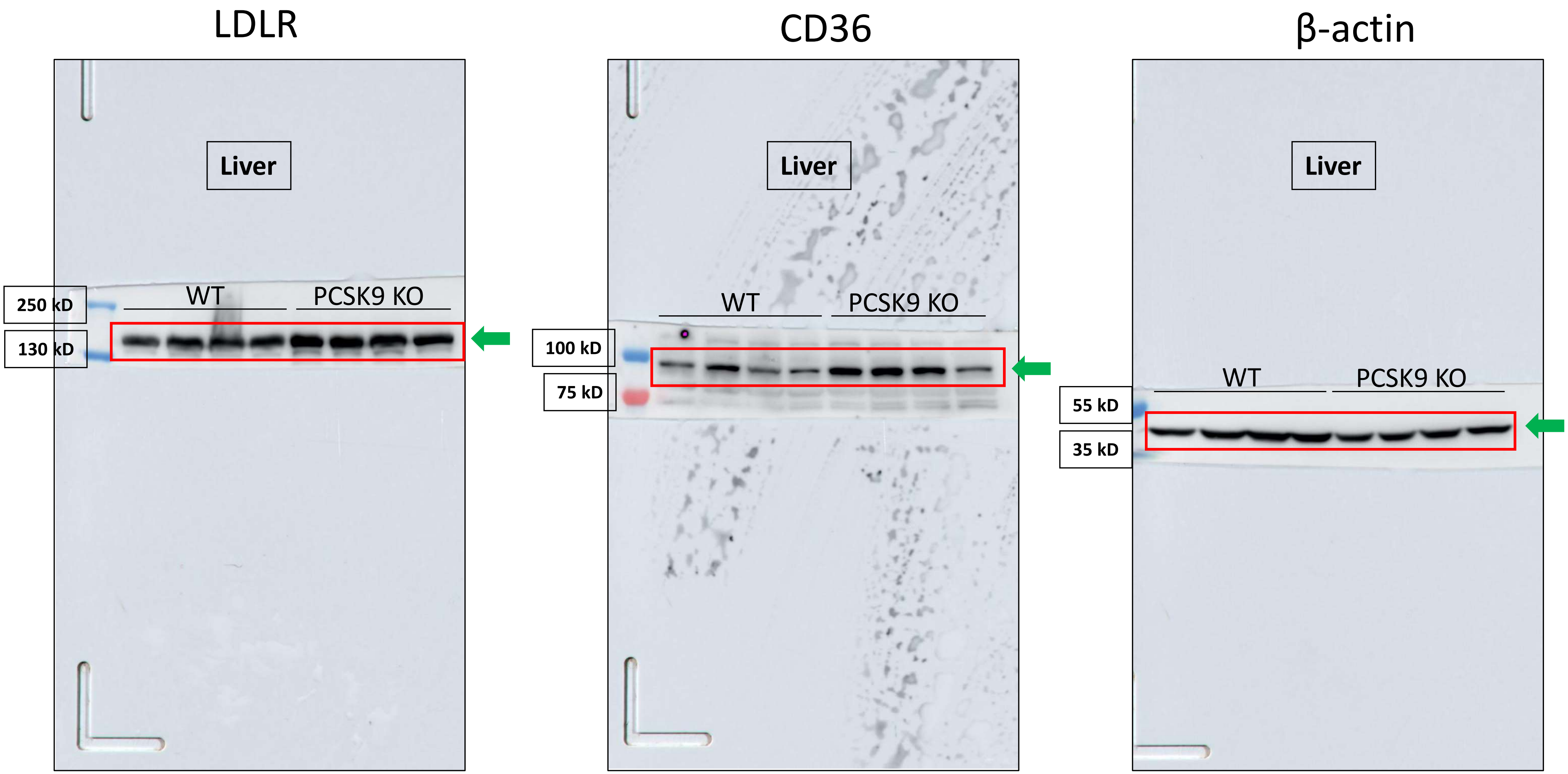

Full unedited blot for Supplementary Figure 7A

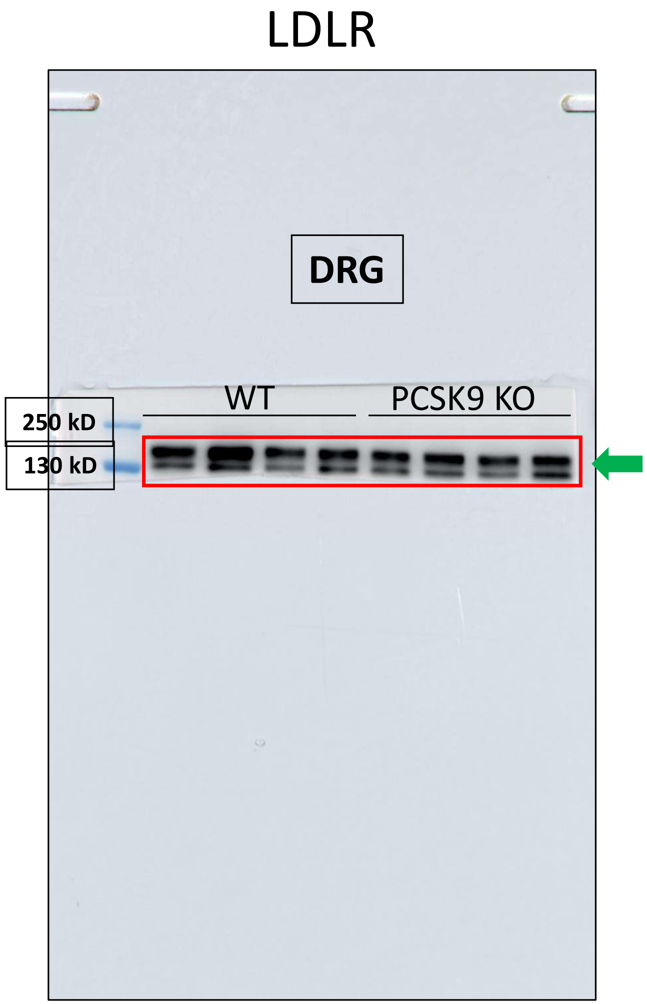

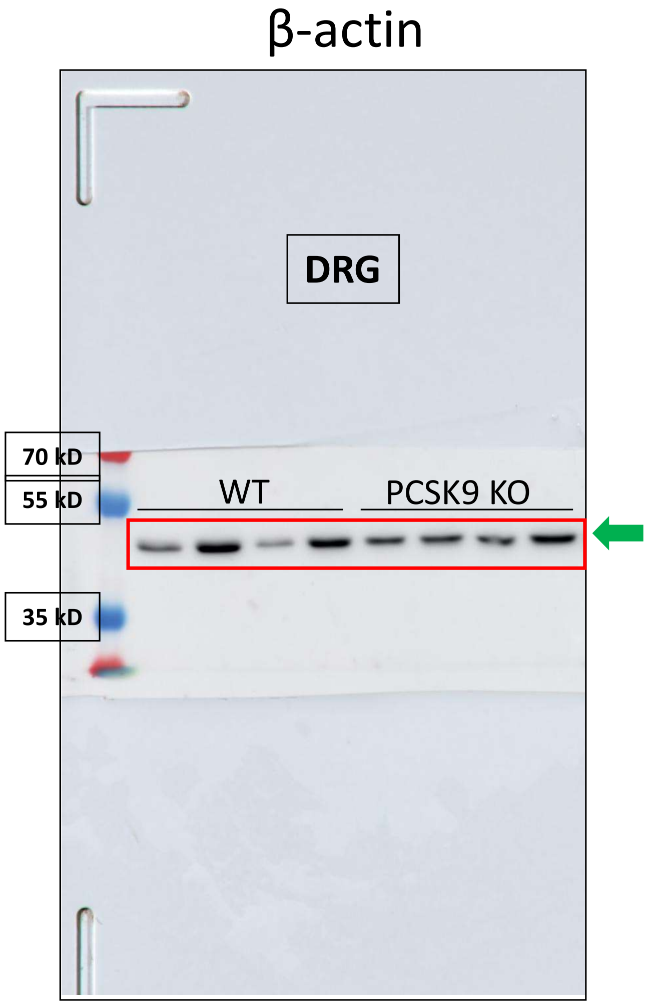

Full unedited blot for Supplementary Figure 7C

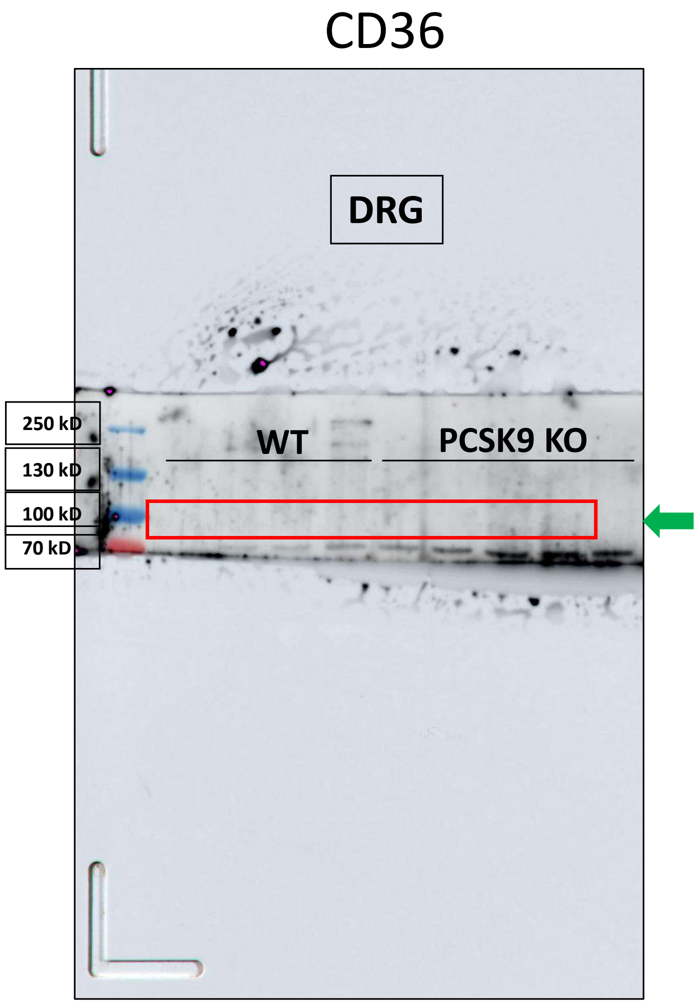

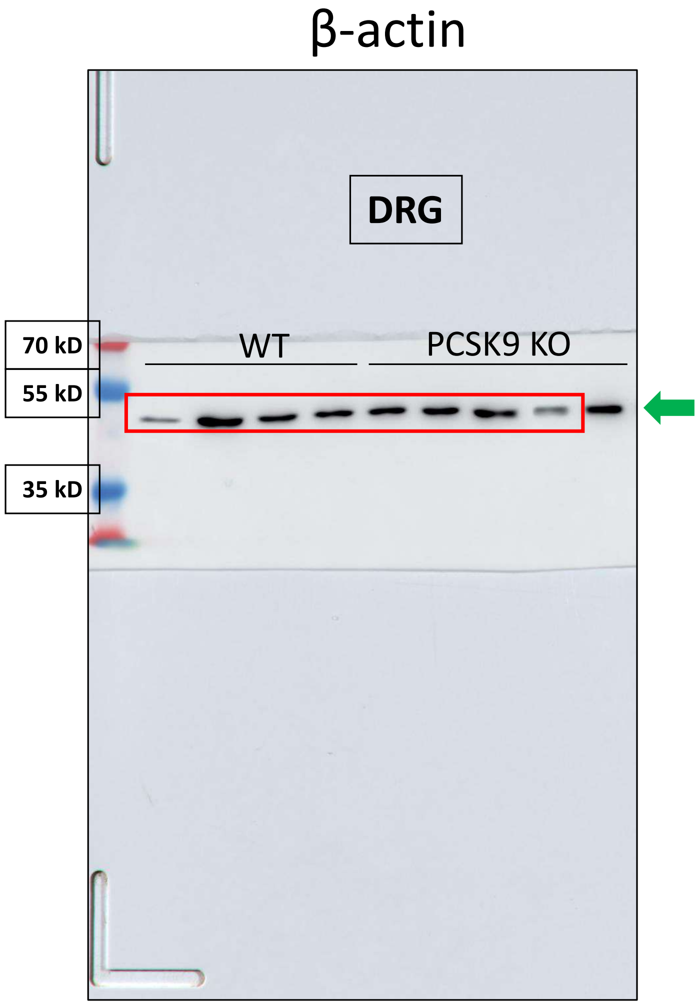

Supplement: Unedited blot and gel images [file jciinsight-10-183786-s218.pdf]
